# Differential Expression of Keratinocyte-Derived Extracellular Vesicle Mirnas Discriminate Exosomes From Apoptotic Bodies and Microvesicles

**DOI:** 10.3389/fendo.2018.00535

**Published:** 2018-09-11

**Authors:** Uyen T.T. Than, Dominic Guanzon, James A. Broadbent, David I. Leavesley, Carlos Salomon, Tony J. Parker

**Affiliations:** ^1^Tissue Repair and Translational Physiology Program, Institute of Health and Biomedical Innovation, Queensland University of Technology, Kelvin Grove, QLD, Australia; ^2^Faculty of Health, School of Biomedical Science, Queensland University of Technology, Brisbane, QLD, Australia; ^3^Wound Management Innovation Cooperative Research Centre, West End, QLD, Australia; ^4^Vinmec Research Institute of Stem Cell and Gene Technology, Vinmec International Hospital, Ha Noi, Vietnam; ^5^Institute of Medical Biology–Agency for Science, Technology and Research, Singapore, Singapore; ^6^Exosome Biology Laboratory, Centre for Clinical Diagnostics, University of Queensland Centre for Clinical Research, Royal Brisbane and Women's Hospital, The University of Queensland, Brisbane, QLD, Australia; ^7^Department of Clinical Biochemistry and Immunology, Faculty of Pharmacy, University of Concepción, Concepción, Chile

**Keywords:** extracellular vesicles, apoptotic body, microvesicle, exosome, microRNA, keratinocytes

## Abstract

Extracellular vesicles (EVs) are mammalian cell-derived nano-scale structures enclosed by a lipid bilayer that were previously considered to be cell debris with little biological value. However, EVs are now recognized to possess biological function, acting as a packaging, transport and delivery mechanisms by which functional molecules (i.e., miRNAs) can be transferred to target cells over some distance. To examine the miRNA from keratinocyte-derived EVs, we isolated three distinct populations of EVs from both HaCaT and primary human keratinocytes (PKCs) and characterized their biophysical, biochemical and functional features by using microscopy, immunoblotting, nanoparticle tracking, and next generation sequencing. We identified 1,048; 906; and 704 miRNAs, respectively, in apoptotic bodies (APs), microvesicles (MVs) and exosomes (EXs) released from HaCaT, and 608; 506; and 622 miRNAs in APs, MVs and EXs released from PKCs. In which, there were 623 and 437 identified miRNAs common to three HaCaT-derived EVs and PKC-derived EVs, respectively. In addition, we found hundreds of exosomal miRNAs that were previously un-reported. Differences in the abundance levels of the identified EV miRNAs could discriminate between the three EV populations. These data contribute substantially to knowledge within the EV-identified miRNA database, especially with regard to keratinocyte-derived EV miRNA content.

## Introduction

Extracellular membrane vesicles (EVs) are secreted by various cell types and can be isolated from body fluids such as breast milk, urine, amniotic fluids, plasma and saliva, as well as from cell-conditioned culture media (1–7). Classically, EVs have been categorized based upon physical parameters in (i) apoptotic bodies (Aps; ~1–5 μm); (ii) microvesicles (MVs; ~100–1,000 nm); and (iii) exosomes (EXs; ~40–100 nm) (8). While APs are products of apoptosis, MVs and EXs are shed and released from cells during normal physiological processes, and in states of disease. During the formation and release process, EVs are assembled as a lipid bilayer membrane encapsulating cell-derived components such as proteins, lipids, organelles and genetic materials including nucleic acids (9–11). In this regard, statistical data in Vesiclepedia report that the EVs database contains 92,897 protein, 32,576 RNA and 584 lipid molecules from 538 studies in 33 species (Vesiclepedia version 3.1 released 9/1/2015, http://www.microvesicles.org/). Moreover, a total of 4,934 microRNA molecules out of 32,576 known RNAs have been found within vesicle cargo, as reported in the literature. Of note, some individual biomolecules are common to most EVs, but different EV populations exhibit variation in their biomolecular composition (10). Molecules are unique for a particular population of vesicle and could be considered as markers of that EV population. For example, annexin V is a marker of APs and MVs (12, 13). Similarly, transmembrane proteins of the tetraspanin family, such as CD9, CD63, and CD81, are known to be markers for exosomes (14). Additionally, EXs from body fluids such as human saliva, plasma and breast milk contain RNAs, but little or no ribosomal RNAs (18S and 28S) (5). Ribosomal RNA subunits 18S and 28S have also been detected in APs, but these ribosomal RNAs were either very rarely detected in MVs or not detected at all (10). These indicate that distinct molecule signatures in three different EV populations can be used to distinguish them.

MicroRNAs (miRNAs) are small non-coding RNAs, generally 19–24 nucleotides long and have been shown to target mRNAs for cleavage or translational repression (15). miRNAs are encoded within and transcribed from the intergenic regions of the genome prior to processing and transport to the cytoplasm. They are then cleaved by the Dicer enzyme and further processed to become a mature miRNA that is incorporated into an RNA-induced silencing complex that is involved in targeted gene suppression (15). Importantly, it is now estimated that miRNAs regulate more than 60% of protein translation via multiple pathways (16, 17). As such, studies of miRNAs are pivotal especially in the context of the emerging EV field. Of relevance, several investigations have revealed the presence of miRNAs in EVs (11, 18, 19). The first paper to report this occurrence described the presence of miRNAs in mast cell-derived exosomes and showed that these miRNAs were transferred horizontally between mast cells (11). Recent studies have also demonstrated that miRNAs can be detected in blood plasma within EXs, MVs and APs derived from cancer cells (19–23). As miRNAs are known to regulate gene expression, it is possible that EV miRNAs may regulate gene expression in recipient cells. However, there have been no studies characterizing the miRNA cargo derived from the three EV populations released from human keratinocytes.

Keratinocytes are the most abundant cell type in the skin and spatially occupy the most basal and superficial layers of the stratified epithelia and as such, serve as the primary barrier between the body's interior and the external environment. The major functions of the skin are: (i) prevent the loss of moisture and heat; (ii) sense and communicate physio-chemical properties of the proximal external environment; (iii) provide a physical barrier to abrasive injury; and (iv) defend against pathogen invasion; and initiate responses to trauma (24, 25). Specifically, foreskin-isolated keratinocytes secrete an extensive catalog of cytoplasmic proteins into exosomes and these proteins, especially stratifin have an MMP-1 stimulatory effect on dermal fibroblasts (26, 27). However, the keratinocyte-derived EVs and their components and functions remain poorly understood. Therefore, this study is the first to isolate APs, MVs and EXs released from keratinocytes in order to analyse their miRNA content and identify the discriminant molecular features of EXs, Aps, and MVs.

## Methods

Extended methods can be found in Supplementary Information.

### Cell culture

Ethical approval for research detailed herein was obtained from: Queensland University of Technology (QUT); Pacific Day Surgery/Brisbane Private Hospital (approval # 1300000063/QUT); Princess Alexandra Hospital (approval # HREC/06/QPAH/91); Uniting Health Care's St. Andrews Hospital and Wesley Hospital (approval # 2003/46).

Epidermal primary keratinocytes (PKCs) were freshly isolated from donor skin and propagated on i3T3 feeder cells using the method of Rheinwald and Green (28). HaCaT cells were purchased from CLS Cell Lines Service GmbH (Eppenheim, Germany) (29). All keratinocyte cultures were maintained at 37°C in a 5% CO_2_/95% air atmosphere, with media changes every 2 days, and subcultured when the cultures became 80% confluent.

### EV production and isolation

PKCs and HaCaT cells were cultured to 80% confluence; the expired media and i3T3 cells were removed and cells were washed with fresh DMEM prior to being incubated for 48 h with serum-free media for EV production. The EV-enriched media (conditioned) media (CM) were collected and centrifuged at 300 × *g* for 10 min to remove cell debris (30).

EV's were isolated using a modification of Crescitelli et al. and Valadi et al. (10, 11). Briefly, CM were gravity filtered through membrane filters with various pore sizes then centrifuged at 3,000 × *g* for 40 min, named AP; at 16,500 × *g* for 1 h at 4°C, name MV; and at 100,000 × *g* for 1.5 h at 4°C, labeled “EX-harvest” pellets. The AP, MV and EX pellets were resuspended and washed in PBS and concentrated again at 3,500 × g for 1 h, term “clean APs”; 16,500 × g for 1 h, term “clean MVs”; and 100,000 × *g* for 1.5 h at 4°C, term “clean EXs,” respectively. “Clean” AP, MV and EX harvest materials were resuspended in PBS (approximately 30 μL) for further experiments. This protocol is summarized in Supplementary Figure 4.

### Protein extraction

A volume of AP, MV, EX, or cell suspension was admixed with an equal volume of extraction buffer in Protein Lo-Bind tubes (Eppendorf) and incubated for 3 min at 95°C. Samples were subsequently sonicated for 5 min at room temperature. The resulting mixtures were centrifuged at 14,000 × *g* for 15 min at 4°C, and the protein supernatant decanted and stored at −20°C until required.

### Immunobloting

Cell lysate and EV preparation were separated electrophoretically with 4–12% SDS-PAGE. Separated proteins were then electrophoretically transferred onto a pure nitrocellulose membrane (Life Technology). Following transfer, the membrane was blocked with 5% skim milk in Tris Buffered Saline/0.01% Tween (TBST). The membrane was then probed overnight at 4°C with diluted primary antibodies against CD9, CD63, CD81, HSP70, and TSG101, or AGO2 (Abcam®) prior to wash and incubation with HRP-conjugated secondary antibody (HAF008, R&D Systems). Antibody binding was detected using the ECL detection solution (Pierce™ ECL Western Blotting Substrate, Thermo Scientific) per manufacturer's instructions and imaged on Curix Ultra UV-G Medical X-ray film (AFGA; Mortsel, Belgium).

### Nanoparticle tracking analysis (NTA)

A volume of 50 μL of EX preparation was diluted using ultra-pure water (Milli-Q® Integral Water Purification System) to a total volume of 500 μL in 1 mL LoBind tubes (Eppendorf). Concentration, size, aggregation and zeta potential of individual EX samples were acquired and analyzed using the NanoSight NS500 with Nanoparticle Tracking Analysis (NTA) 3.0 software (Malvern, Worcestershire, UK).

### Transmission electron microscopy (TEM) and confocal microscopy

EV samples were fixed with saline buffered 4% paraformaldehyde, and deposited onto Formvar-carbon coated grids (Ted Pella, Inc., Redding CA). EV samples were washed eight times with PBS, stained with uranyl-oxalate and over-layered with methylcellulose. Imaging was performed using a JEOL 1400 Transmission Electron Microscope (TEM; JEOL Ltd., Tokyo, Japan) at 80 kV.

Clean AP pellets were resuspended in 100 μL of 1 × binding buffer (Annexin V-FITC Apoptosis Detection Kit, Abcam, Cambridge, UK) and probed for phosphatidyl serine (Annexin V-FITC) and nucleic acids (propidium iodide) per the manufacturer's instructions. Approximately 5 μL of the AP suspension was applied to a glass slide, observed and photographed (60× objective) under epifluorescence using a FITC and rhodamine dual filter using a Leica TSC SP5 (Leica Microsystems, Germany).

### Total RNA extraction

Total RNA was extracted using the Trizol™ method following the manufacturer's protocol (31, 32). Briefly, Trizol™ reagent (Thermo Fisher Scientific) was admixed to either whole cell suspension, APs, MVs or EXs (9 part Trizol: 1 part cells/vesicles). The Trizol-vesicle solution was triturated or vortexed to ensure vesicle lysis prior to addition of MgCl_2_ solution (Sigma) and chloroform. Each mixture was vortexed vigorously, incubated at room temperature (RT) for and centrifuged prior to transfer of the aqueous phase to fresh 2 mL micro tubes. Then, isopropanol was added and the samples were repeatedly inverted and incubated at RT. The tubes were then incubated at −20°C for 1 h (or overnight). Following incubation, the samples were centrifuged at 12,000 × *g* for 10 min at 4°C to collect RNA pellet. The RNA pellets were washed in RNAse-free 75% ethanol twice prior to removal of the supernatants and allowing the RNA pellets to air dry. Finally, the RNA was resuspended in 10–20 μL RNase-free water (Invitrogen; depending on the size of the RNA pellets).

Vesicle-derived RNA was quantified and evaluated for quality (as described in the Supplementary Material). RNA samples of sufficient quality were initially subjected to qRT-PCR to confirm the presence of miRNAs prior to subjecting RNA to deep sequencing using the Illumina® NextSeq500 system (below).

### Sequencing microRNAs using Illumina® next Seq500

A representative RNA library from each EV preparation was constructed using the Illumina® TruSeq® Small RNA Library Prep Kit as per the manufacturer's instructions. Initially, the total RNA was ligated to RNA adapters prior to a conduction of reverse transcription. The resulting cDNA was amplified by PCR using primers designed to anneal to the ends of the adapters. The amplified PCR products from this stage were referred to as the small RNA library which was subsequently purified by gel electrophoresis. The small RNA library was eluted in 200 μL pure water by incubation overnight with shaking and then validated using a Bioanalyzer. The resulting cDNA library was diluted to 2 nM using a solution of 10 nM Tris–HCl, pH 8.5 and 0.1% Tween 20 prior to loading onto an Illumina chip and sequenced using an Illumina® NextSeq500. RNASeq data is available at GEO under accession number GSE106453.

### miRNA identification and statistics

Sequencing using the Illumina® Next Seq500 resulted in a FASTQ file.Each raw FASTQ file was curated as follows. Index and adaptor sequences were removed and trimmed to 28 nucleotides using the TagCleaner program (http://tagcleaner.sourceforge.net/index.html, version 0.16) and FASTX-Toolkit program (http://hannonlab.cshl.edu/fastx_toolkit/index.html, version 0.0.13) respectively. “Cleaned” nucleotide data were interrogated using miRDeep2 software and candidate sequences aligned to the human genome (hg19), miRNAs of identified and relative quantity calculated.

The identified miRNAs and their raw counts were further analyzed using the DESeq2 package (version 1.10.1). Each candidate miRNA sequence was filtered, normalized and tested for differential expression using a negative binomial generalized linear model (33). A Wald test was applied to calculate statistical significance and adjusted for multiple testing using the Benjamini and Hochberg procedure (33). Results were considered different where an adjusted *p*-value < 0.01 was determined between groups. Graphical illustrations and heatmaps were produced using the R statistical environment (R version 3.2.2, last update 14/8/2015) with gplots package (version 2.17.0) (34).

### Exocarta database

Candidate miRNAs found to be associated with individual EV samples were interrogated against the curated ExoCatra database (exocarta.org, version 5, released on 29 July 2015) containing a total of 2,766 miRNAs (*Homo sapiens*).

### Bioinformatic analysis

In order to analyse the miRNA-target gene interactions, EV miRNA target genes were identified by submission of the miRbase IDs for the EV miRNAs to Cytoscape (version 3.2.1) and searching against the miRTarBase database (accessed 5/1/2016) using the CyTargetLinker tool in Cytoscape. An interaction network of the miRNAs and their target genes was created and exported for further interpretation. The target genes then were analyzed for biochemical pathways using Panther (version 12) (35).

### Data presentation and statistical analysis

GraphPad Prism 6 for MacBook (GraphPad Software, La Jolla California USA, www.graphpad.com) and the R Environment for Statistical Computing version 3.2.2. were used to produce graphs and to perform statistical analysis. Data are presented as the Mean ± SD and statistical significance was determined by Wald test, using the R program or Student's *t*-test, as indicated and statistical significance was accepted at a *p*-value of <0.05.

## Results

### Three EV populations released from keratinocytes exhibit different morphology, size and protein markers

**Figure 1:**
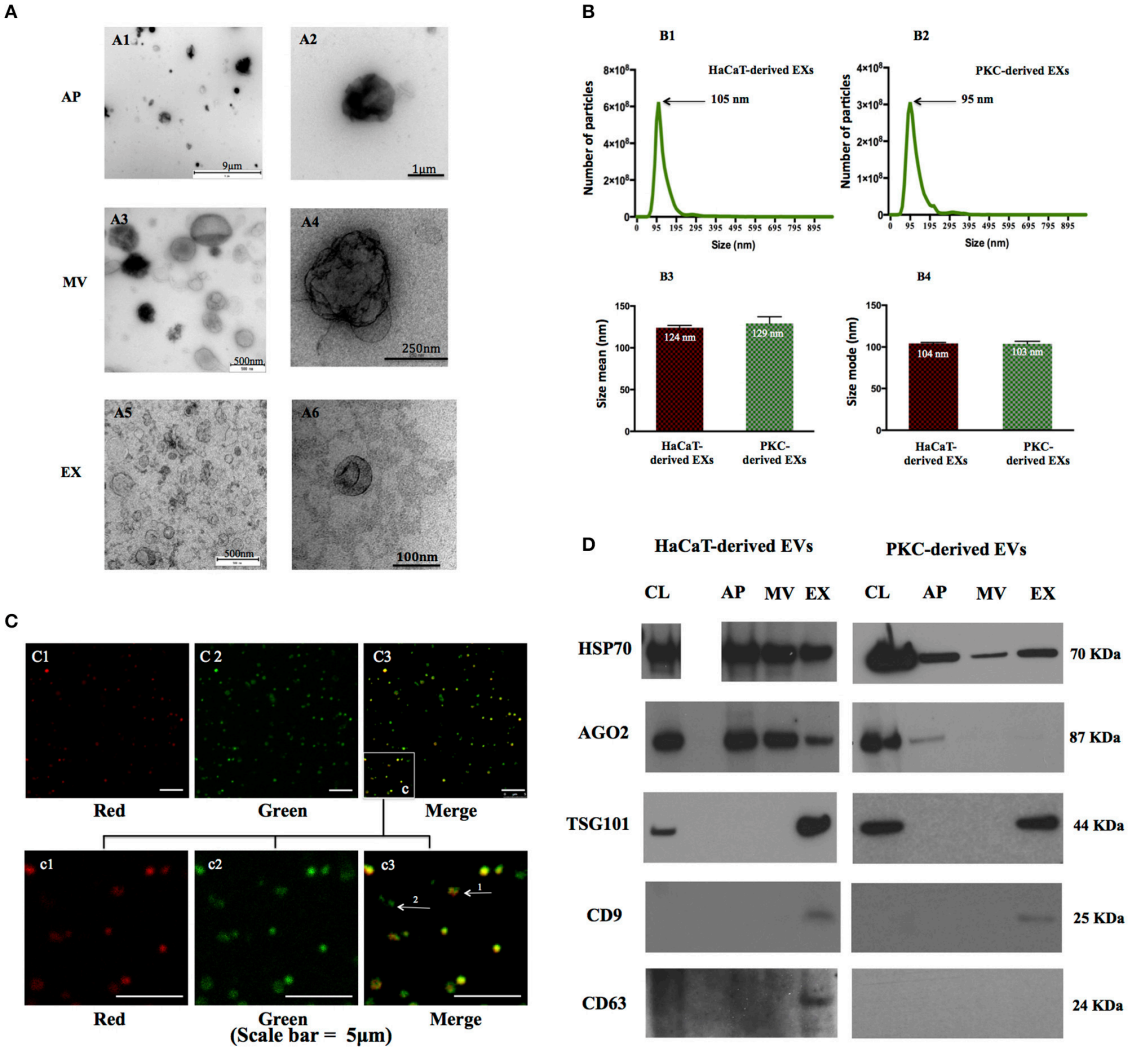
EV populations secreted by human keratinocytes exhibit distinct physio-chemical properties. EV were isolated and characterized from keratinocyte cultures. **(A)** Morphology of three EV populations was revealed by TEM with a magnification range of 50–2,000,000×. Four independent biological samples were analyzed (HaCaT cells at passage 49, 50, primary keratinocytes isolated from patient # 288, 300). Representative images are of EVs released from HaCaT passage 50). **(B)** The size distribution of exosome populations were analyzed the NanoSight NS 500 system. Three biological replicates were analyzed for each experiment (HaCaT cell line at passage 49, 50, and 51; PKC patients: #296 at passage 1, #325 at passage 1, and #366 at passage 2). Error bars indicate SD; statistical significance was determined using Student's *t*-test. **(C)** AP fractions were examined and photographed by confocal microscopy using a 60× objective. Vesicles were probed with propidium iodide (PI) for DNA (red) and Annexin V-FITC for phosphytidyl serine (PS; green). Notably a sub-population of APs reacted positively with probes for both nucleic acid fragments and PS (arrow 1), whereas other APs reacted positively for PS only (arrow 2). Replicates from HaCaT passage 50, HaCaT passage 53 and PKC isolated from donor # 288 are illustrated; Images are representative for APs released from HaCaT cells passage 53. **(D)** Proteins enriched in EVs were detected in EVs derived from both HaCaT and PKCs. Representative images of immunoblots of characteristic proteins (TSG 101, AGO2, CD9, and CD63) and 363 (HSP70) identify EVs released from HaCaT passage 50 and PKC passage 1 from patient 306. CL, cell lysate; AP, apoptotic bodies; MV, microvesicles; EX, exosomes.

Three distinct populations of EVs were isolated from media conditioned for 48 h by human primary epidermal keratinocytes (PKCs) and HaCaT cells using a modified differential centrifugation protocol (10, 36). We analyzed the morphological characteristics of individual vesicles from each donor and EV population using TEM (Figure 1, Supplementary Figure 1). As previously described (10, 37), we found that vesicles from the AP fraction were typically larger than 1 μm; the MV fraction were irregular and 300 nm to 700 nm in size; and the EX fraction were relatively homogenous at 50 nm to 120 nm with a cup-shaped morphology (Figure 1A). However, we also observed a population of vesicles smaller than 1 μm in the AP fractions and also a few larger (~200 nm diameter), cup-shaped vesicles in the EX fractions (Figures 1A5,A6). Low level EV contamination of AP preparations may have resulted from a sub-population of EVs with low buoyant density that pelleted at low *g* forces.

The nano-scale dimensions of EVs present a challenge for accurate characterization. Therefore, we adopted Nanoparticle Tracking Analysis (NTA), in which the Stokes-Einstein equation is applied to light scattering and Brownian motion to calculate the hydrodynamic parameters of individual particles, which in this case were human keratinocyte-derived EX populations. EXs prepared from human keratinocytes were found to range in size from ~60 nm to ~220 nm in size (Figures 1B1,B2). More specifically, the mean size of PKC-derived EXs was 129 nm while the mean size of HaCaT-derived EXs was 124 nm (Figure 1B3). The size mode of HaCaT-derived EXs and primary keratinocyte-derived EXs was equivalent at 104 and 103 nm, respectively (Figure 1B4).

In contrast to MVs and EXs, APs contain fragments of nuclear DNA and exhibit phosphatidylserine (PS) on the extracellular surface of the vesicle membrane following translocation from the luminal surface during apoptosis and EV shedding (38, 39). Immunofluorescent analysis, utilizing the affinity of Annexin-V for PS and Propidium Iodide (PI) for the detection of nucleic acids, was performed to identify APs in the keratinocyte conditioned media (Figure 1C). While, many of the large EVs that were stained with PI and Annexin-V appeared to have a spherical morphology approximately 1 μm in diameter, others were stained with Annexin-V only and were typically smaller with an irregular morphology (Figures 1C1–C3). This may suggest a contamination of MVs in the AP fraction, or that some APs, literately, do not contain DNA material.

We also found that the EVs in the MV preparations reacted with Annexin-V only and failed to react with PI suggesting that MVs were either intact, excluding PI, or were free from nucleic acids (Supplementary Figure 2).

In order to more comprehensively characterize each EV population, we performed immunoblots for protein markers previously associated with EVs; namely: tetraspanins (CD9, CD63); TSG101; and the intracellular proteins, AGO2 and HSP70 (9, 40, 41). Although we were careful to ensure that total protein from the cell lysates were utilized as controls for protein enrichment some proteins may or may not be expressed in parental cells while they are expressed in the corresponding EVs, or *vice versa* (41). The data revealed that three out of the five markers investigated, including HSP70, TSG101, AGO2 (PKCs only), were detected in parental cell controls and each of the corresponding EVs (Supplementary Figure 3). However, CD9 and CD63 were detected in EXs, but not in their respective parental cells. As alluded to above, CD63 was only detected in HaCaT-derived EXs while no immunoreactive band was detected in in PKC-derived EXs (Figure 1D).

Taken together, these data indicate that three EV populations, including APs, MVs and EXs, were released from both HaCaT and PKCs in 2D culture. These vesicles exhibited individual characteristics in term of morphology, size and bio-molecular markers.

### Keratinocyte-derived EV miRNA profiles

In order to characterize the miRNA contained within EVs released from keratinocytes, a small RNA next generation sequencing approach was performed on the three EV populations and their parental cells using the Illumina® NextSeq500 platform. The total raw reads were filtered by discarding reads <16 nucleotides in length, prior to alignment with the human genome (hg19). The cells, APs, MVs and EXs exhibited the number of read count and identified miRNAs between the biological replicates (Tables 1, 2). Additionally, the large number of identified miRNAs indicated the enrichment of miRNAs in the three EV populations isolated from keratinocyte cultures (Tables 1, 2). All identified miRNAs with greater than one count were used for further analysis.

**Table 1:** Summary of small RNA sequencing from HaCaT samples.

**Biological repeats**	**HaCaT cells**		**AP**		**MV**		**EX**	
	**1**	**2**	**1**	**2**	**1**	**2**	**1**	**2**
Total raw reads	28,059,972	96,532,400	87,922,560	111,419,044	59,342,340	85,436,024	9,550,052	12,994,788
Filtered reads	6,136,227	18,013,320	21,125,475	25,768,444	14,295,262	19,905,748	2,017,355	2,244,521
Number of alignments to Hg19	3,819,215	7,963,825	4,493,879	8,962,780	3,401,620	4,509,155	1,040,299	1,438,313
Percentage of alignments^a^	62.24%	44.21%	21.27%	34.78%	23.79%	22.65%	51.56%	64.08%
Number of reads for identified miRNAs	574,748	2,205,272	1,134,051	4,667,465	924,525	1,661,704	295,556	439,718
Number of identified miRNAs (%^b^)	861 (33%)	1,107 (43%)	929 (36%)	1,217 (47%)	879 (34%)	1,022 (39%)	727 (28%)	799 (31%)
Number of miRNA with read = 1	233	230	183	240	226	223	194	205

a *Calculated as percentage of filtered reads*.

b *Calculated as percentage of identified miRNAs to known mature miRNAs (miRBase release 21, 2,588 mature miRNAs)*.

**Table 2:** Summary of small RNA sequencing from PKC samples.

**Biological repeats**	**PKC cells**				**AP**				**MV**				**EX**			
	**1**	**2**	**3**	**4**	**1**	**2**	**3**	**4**	**1**	**2**	**3**	**4**	**1**	**2**	**3**	**4**
Total raw reads	47,418,680	139,148,800	95,328,248	155,126,328	41,054,732	7,932,424	10,474,160	12,041,344	3,802,540	5,365,988	465,348	4,373,356	2,035,032	3,190,492	12,259,328	14,348,924
Filtered reads	10,405,473	25,269,296	15,491,519	29,424,103	9,990,189	1,782,028	2,290,890	2,792,236	881,221	1,082,908	66,724	846,020	411,108	528,624	2,384,147	2,467,907
Number of alignments to Hg19	7,836,109	12,952,004	5,006,563	16,064,246	3,399,071	410,115	644,360	741,333	313,934	473,642	20,131	351,979	231,451	299,923	487,323	1,679,084
Percentage of alignments^a^	75.30%	51.25%	32.31%	54.59%	34.02%	23.01%	28.12%	26.55%	35.62%	43.74%	30.17%	41.60%	56.30%	56.74%	20.44%	68.04%
Number of reads for identified miRNAs	921,682	910,636	200,980	2,442,668	299,536	21,150	32,568	121,341	83,725	82,194	3,322	74,379	102,136	93,287	25,918	225,736
Number of identified miRNAs (%^b^)	940 (36%)	1,106 (43%)	705 (27%)	1,336 (52%)	682 (26%)	346 (13%)	445 (17%)	559 (22%)	476 (18%)	505 (20%)	214 (8%)	510 (20%)	511 (20%)	528 (20%)	394 (15%)	703 (27%)
Number of miRNA with read = 1	202	266	185	336	180	97	126	150	123	135	74	168	137	130	109	186

a *Calculated as percentage of filtered reads*.

b *Calculated as percentage of identified miRNAs to known mature miRNA (miRBase release 21, 2,588 mature miRNAs)*.

### Detection of common and unique miRNAs between the three EV subtypes

**Figure 2:**
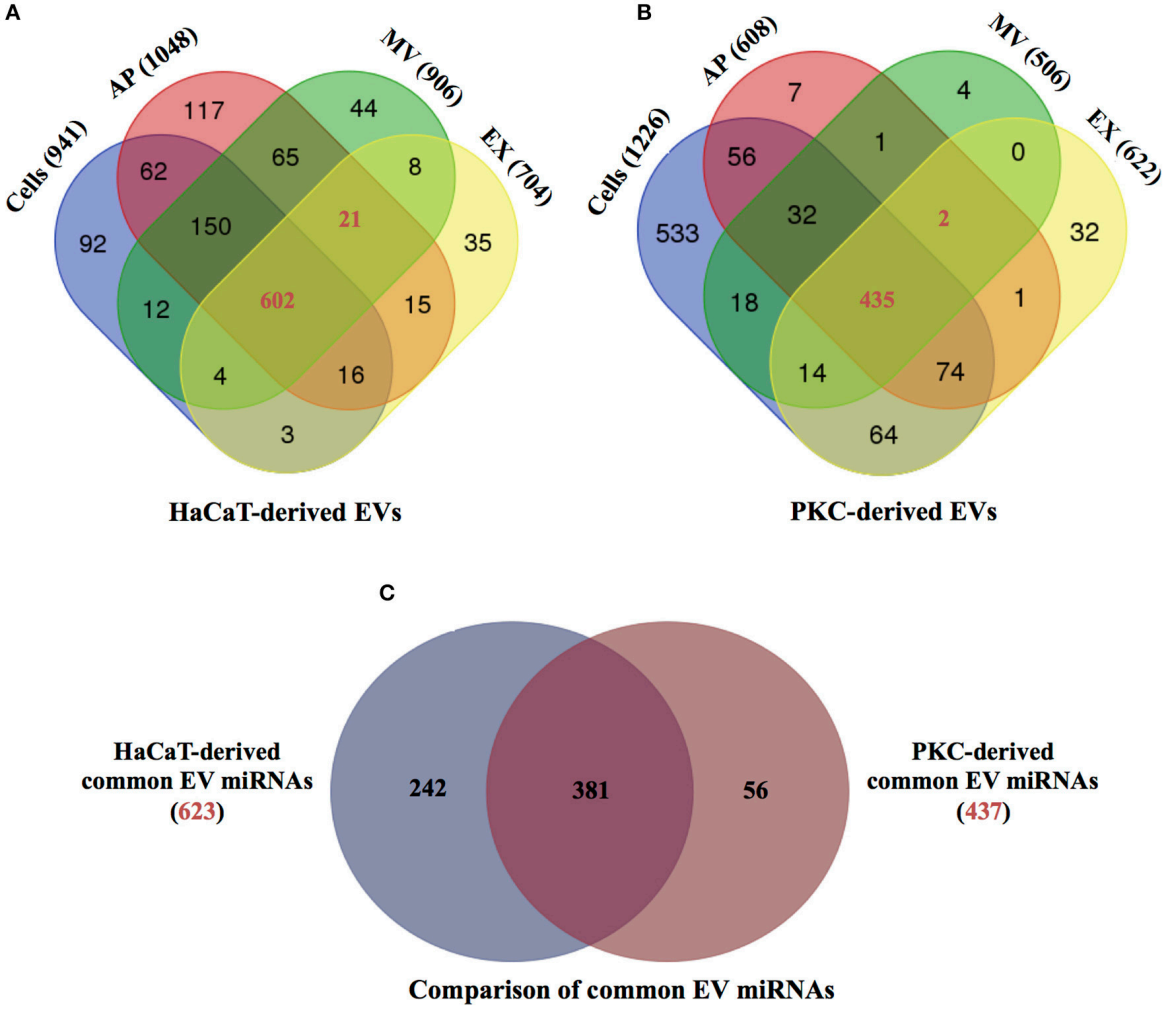
miRNA distribution between parental HaCaT and primary keratinocytes and respective EV populations. The distributions of the identified miRNAs having greater than one count, from **(A)** HaCaT-derived EVs and HaCaT cells (*n* = 2 independent biological replicates); and **(B)** primary keratinocyte-derived EVs and primary keratinocytes (*n* = 4 independent biological replicates). **(C)** The distribution of common miRNAs from HaCaT-derived EVs (red number from A, 623 miRNAs) and primary keratinocyte-derived EVs (red number from B, 437 miRNAs; Supplementary Table 2). PKC: primary keratinocytes. Venn diagrams were generated using web-base Venn draw tool (http://bioinformatics.psb.ugent.be/webtools/Venn/).

In order to reveal the degree of overlap in the miRNA population between the different EV subtypes and their parental cells, the individual miRNA species identified with more than one count for each cell type and their respective EVs were subjected to comparative analysis (Figure 2). The results indicated that there were 941, 1,048, 906, and 704 miRNAs identified in parental HaCaT cells, HaCaT-derived APs, MVs and EXs, respectively (Figure 2A). Furthermore, all but 92 of the 941 miRNAs identified in the HaCaT parental cells were also identified in one or more of the HaCaT-derived EV populations, while 623 miRNAs were common to the three populations of HaCaT-derived EVs (Figure 2A). Of the PKC derived miRNAs, 1,226, 608, 506, and 622 miRNAs were identified in parental PKCs, PKC-derived APs, MVs, and EXs, respectively (Figure 2B). A common set of 437 miRNAs were observed between the three EV populations in addition to unique miRNAs for each PKC-derived EV population (Figure 2B).

In order to determine if there were any differences in the miRNA composition associated with EV cellular origin, the 623 common HaCaT-derived EV miRNAs (Figure 2A) and the 437 miRNAs common to PKC-derived EVs (Figure 2B) were compared. The results showed that while there were 381 miRNAs common to EVs derived from both HaCaT and PKCs, 242 and 56 miRNAs were unique to HaCaT- and PKC-derived EVs, respectively (Figure 2C). Among the common HaCaT- and PKC-derived EV miRNAs, miRNA families detected with more than five members were observed, including: hsa-let-7 miRNA; hsa-miR-181; hsa-miR-100; hsa-miR-30; hsa-miR-125; and hsa-miR-27 (Supplementary Table 1).

### Correlation of miRNA population between parental keratinocytes and their respective vesicles

**Figure 3:**
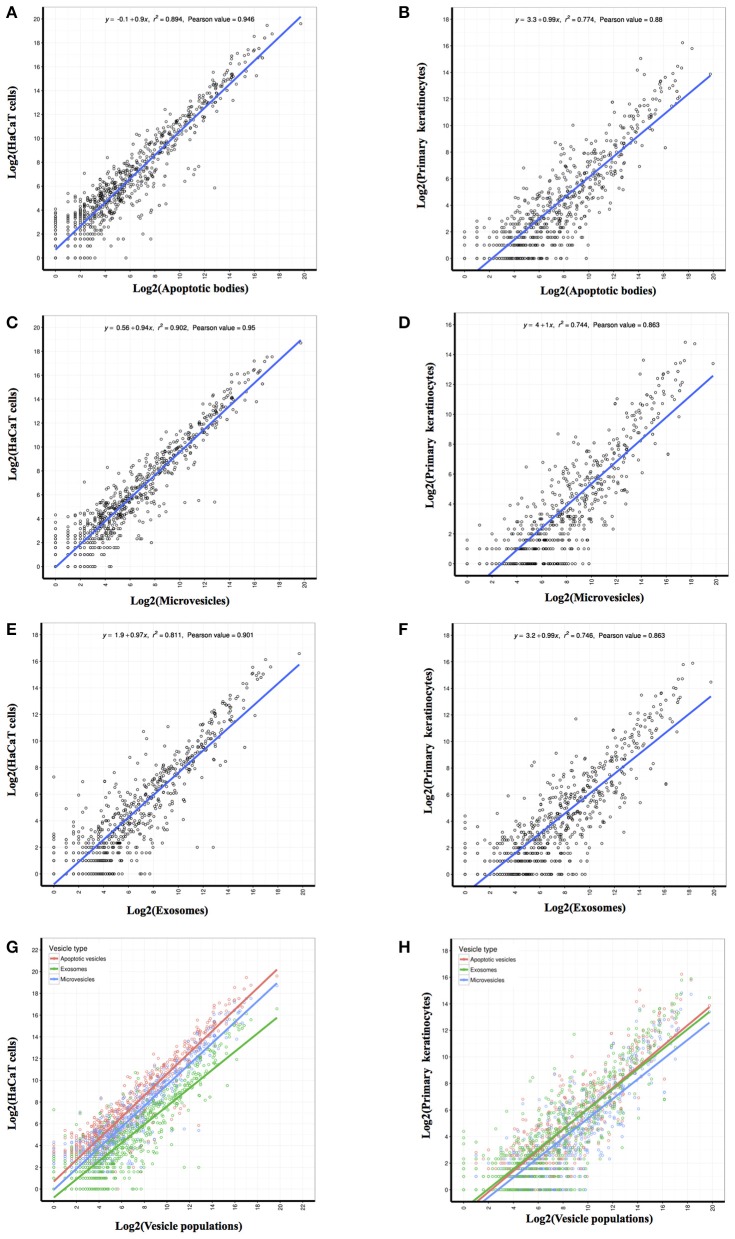
Correlation coefficient analysis of identified miRNAs revealed the linear relationship between each EV population and its parental cell. The abundance value for each identified miRNA was log2 transformed prior to performance of the person correlation analysis. Pearson correlation coefficients were generated between the miRNA identified from HaCaT cells and PKC parental cells and those identified in: **(A,B)** Apoptotic bodies; **(C,D)** Microvesicles; and **(E,F)** Exosomes. Merge of Pearson correlation of miRNA identified in each EV population and miRNA identified in **(G)** HaCaT cells and **(H)** PKCs.

In order to derive a better understanding of the relationship between vesicles and their parental cells, a correlation coefficient analysis of the sequence data from the identified miRNAs showed a moderate correlation of miRNA contents between vesicles and their respective parental cells (Figure 3). We also found that the Pearson correlation coefficients between the miRNA quantified in HaCaT parental cells and their vesicle populations (Pearson values: 0.946 between cells and APs, 0.95 between cells and MVs, and 0.901 between cells and EXs; Figures 3A,C,E) were greater than those for PKCs and their corresponding vesicle populations (Pearson value: 0.88 between cells and APs, 0.863 between cells and MVs, and 0.863 between cells and EXs; Figures 3B,D,F). These data indicate that the population of miRNA deposited into PKC-derived vesicles are relatively different to those that remain in PKC cells themselves. This was in contrast to the miRNA deposited into HaCaT-derived vesicles which exhibited a comparatively more similar profile to the miRNA within their parental HaCaT cells.

### Previously un-reported miRNAs derived from EXs

**Figure 4:**
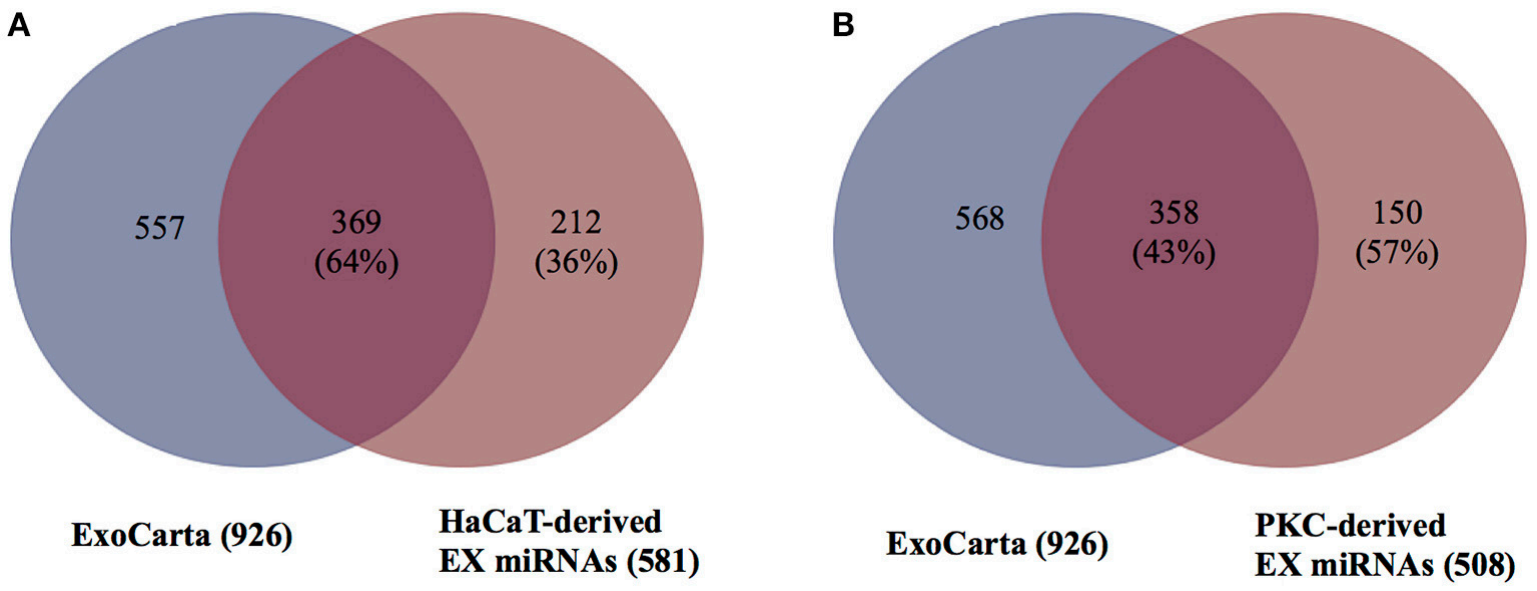
HaCaT and primary keratinocyte derived EXs contain miRNAs previously un-reported as EX cargo. In order to determine the number of previously un-reported miRNAs, the EX miRNAs from the ExoCarta database and the identified EV miRNAs were modified by removal of the lettered suffix indicating 5′ and 3′ of the precursor miRNAs to ensure that the miRNA name was in a compatible format prior to analysis. The 926 miRNAs in the ExoCarta database were compared to: **(A)** HaCaT-derived EXs; and **(B)** primary keratinocyte-derived EXs. The analysis and Venn diagram were generated using the R program.

To determine if any of the detected exosomal miRNAs from either HaCaT cells or PKCs had not been previously reported, the list of miRNAs detected in the keratinocyte-derived EXs were compared to miRNAs from the ExoCarta database (Version 5, released on 29 July 2015, Supplementary Figure 7). The comparison revealed that 369 miRNAs out of 581 miRNAs from HaCaT derived EXs (64%) had been previously described in the ExoCarta database (Figure 4A). In addition, for PKC-derived EXs, 358 miRNAs out of 838 miRNAs (43%) had been previously reported in the ExoCarta database (Figure 4B). Therefore, 212 and 150 miRNAs from HaCaT and PKC derived EXs, respectively, had not been previously described in the ExoCarta database, and thus had not been previously identified as EX cargo (Supplementary Table 2).

### EV miRNA cargo discriminate exosomes from apoptotic bodies and microvesicles

**Figure 5:**
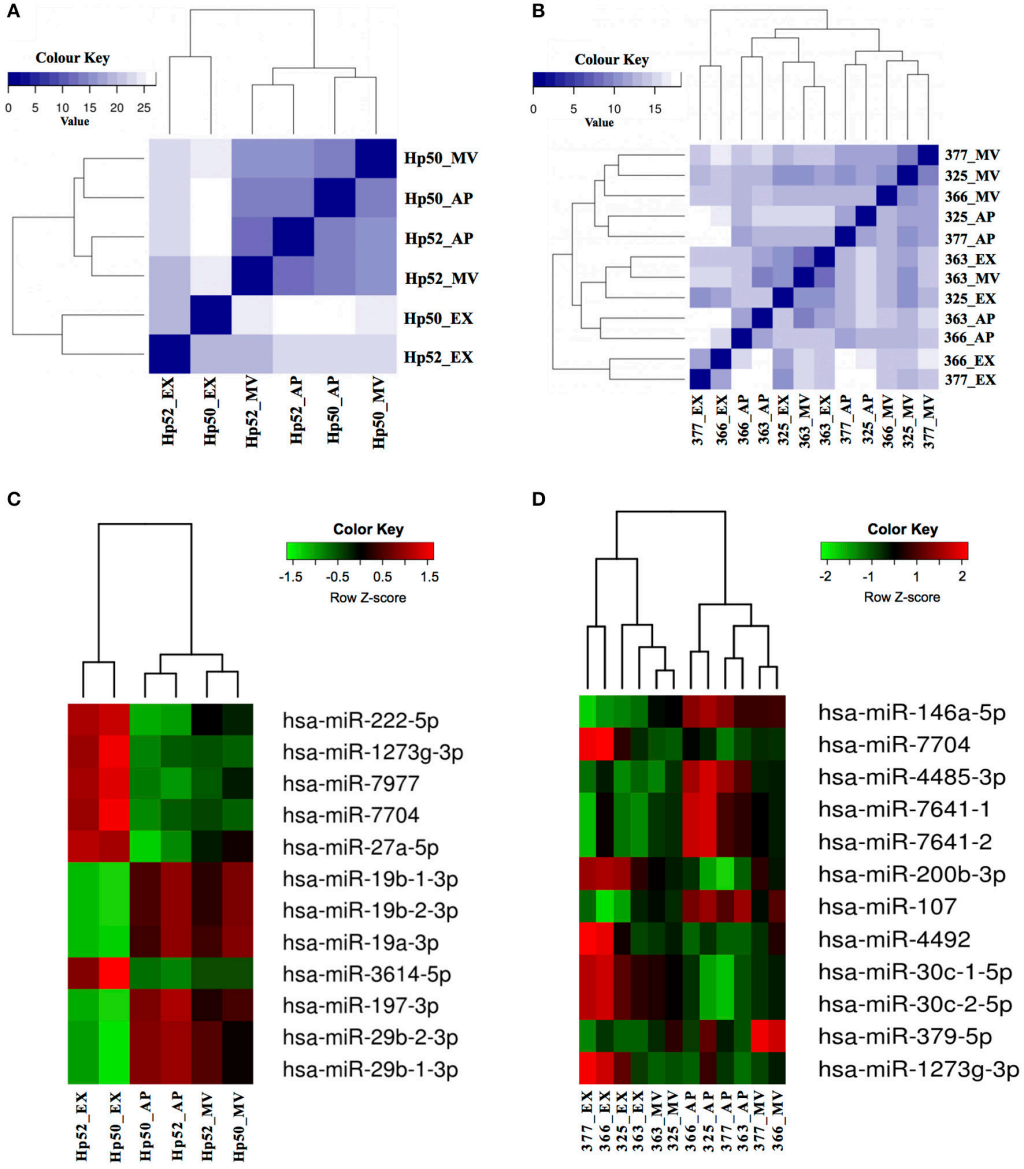
EV derived miRNAs can be used to distinguish between HaCaT and PKC EV sub-populations. The identified EV miRNAs were examined for correlations between the three EV populations derived from **(A)** HaCaT cells (*n* = 2 HaCaT p50 and p52); and **(B)** PKCs (*n* = 4 independent biological replicates) using Euclidean distance analysis, based on the total miRNA profile. The color scale indicates the degree of correlation between EVs, in which a dark blue color indicates greater correlation and lighter color scale indicates less correlation. Subsequently, hierarchical clustering of the 12 most statistically significant and differentially expressed miRNA from **(C)** HaCaT and **(D)** PKCs was performed and presented as a heatmap. The color scale indicates the abundance level of miRNAs, which were ranked based on *p*-values with the most significant first in the list. Hp50 and Hp52 indicate HaCaT cells were at passage 50 and 52; while 377, 366, 325, and 353 indicate skin donor numbers. AP, apoptotic bodies; MV, microvesicles; EX, exosomes. Heat maps were generated using R program (v 3.2.2).

APs, MVs and EXs are formed and released from cells through different pathways and carry distinct molecular cargo (42). The degree of correlation between the miRNA within the three EV populations described herein was examined by Euclidean distance analysis using the total miRNA profile. These data indicated that the miRNA component of APs and MVs were more closely related compared to the miRNAs derived from EXs (Figures 5A,B). A clearer discrimination was observed in the total miRNA counts of EVs from HaCaT cells compared to EVs from PKCs. Specifically, HaCaT p52 and HaCaT p50 derived EXs clustered as a group, which was separate from APs and MVs (Figure 5A). Additionally, PKC derived EXs released from donors # 325 and # 363 were classified together with MVs released from donor # 363, whereas EXs released from donors # 377 and # 366 were classified together (Figure 5B).

Analysis of the miRNA expression levels revealed that there were 73 and 16 miRNAs that exhibited significant differences (*p* < 0.01; Wald test) between the three EV populations released from HaCaT and PKCs, respectively (Supplementary Table 3). Similar to the Euclidean distance analysis, hierarchical clustering of the 12 most significant and differentially expressed miRNAs showed that HaCaT derived EVs clearly clustered into their respective groupings (Figure 5C). However, the 12 most significant and differentially expressed miRNAs from PKC derived EVs exhibited apparent donor based influence on the clustering. Specifically, the EX derived miRNAs from donor #363 clustered more closely with MV derived miRNAs from the same donor (Figure 5B). This potentially reflects a relationship between the EV derived miRNAs and the physiological condition of the parental cells.

Of the most significant HaCaT-derived EV miRNAs, two main groups exhibited inverse expression levels between APs, MVs, and EXs. The six miRNAs: hsa-miR-222-5p; hsa-miR-1273g-3p; hsa-miR-7977; hsa-miR-7704; hsa-miR-27a-5p; and hsa-miR-3614-5p were more abundant in EXs compared to either MVs or APs (Figure 5C). Conversely, hsa-miR-19b-1-3p; hsa-miR-19b-2-3p; hsa-miR-19a-3p; hsa-miR-197-3p; hsa-miR-29b-2-3p; and hsa-miR-29b-1-3p were most abundant in APs and MVs compared to EXs (Figure 5C). Similarly for PKC-derived EVs, the 12 most significant miRNAs were clustered into three distinct groups based on their differential expression. The first group included: hsa-miR-146a-5p; hsa-miR-4485-3p; hsa-miR-7641-1; hsa-miR-7641-2; and hsa-miR-107, which were more abundant in APs, generally lower in abundance in MVs, and had the lowest abundance in EXs (Figure 5D). The second group included: hsa-miR-7704; hsa-miR-200b-3p; hsa-miR-4492; hsa-miR-30c-1-5p; hsa-miR-30c-2-5p; and hsa-miR-1273g-3p, which were most abundant in EXs, lower in MVs and lowest in APs (Figure 5D). The third group only consisted of hsa-miR-379-5p, which was more abundant in MVs, compared to APs and EXs (Figure 5D). Overall, the expression of these miRNAs in PKC derived EVs appeared to be more variable between donors, compared to the miRNA expressed in HaCaT derived EVs (Figures 5C,D).

### Bioinformatic analysis of miRNAs with greater abundance in select EV groups reveal unique target genes and biochemical pathways

In order to gain further insight into the potential functions of the various EV populations, the miRNAs that were differentially expressed between APs, MVs, and EXs were analyzed with respect to their target genes. As described above the miRNA content of APs and MVs was distinct from that of EXs. Therefore, the miRNAs with the greatest abundance in APs and MVs were analyzed together as a group, whereas the miRNAs with the greatest abundance in EXs were analyzed separately. The results showed that the miRNAs that were more abundant in HaCaT-derived APs and MVs, including hsa-miR-19b-1-3p, hsa-miR-19b-2-5p, hsa-miR-19a-3p, hsa-miR-197-3p, hsa-miR-29b-2-3p, and hsa-miR-29b-1-3p, are collectively involved in the regulation of 917 genes (Supplementary Figure 5A). The miRNAs that are more abundant in HaCaT-derived EXs, including hsa-miR-222-5p, hsa-miR-1273g-3p, hsa-miR-7977, hsa-miR-7704, hsa-miR-27a-5p, and hsa-miR-3614-5p, were found to be known to regulate 1149 genes (Supplementary Figure 5B). There were 126 genes known to be regulated by both miRNA groups, in addition to 791 genes and 1023 genes thought to be uniquely regulated by HaCaT-derived APs and MVs, and HaCaT-derived EXs, respectively. With regards to PKC-derived EVs, the miRNAs that were more abundant in APs and MVs including hsa-miR-146a-5p, hsa-miR-4485-3p, hsa-miR-7641-1, hsa-miR-7641-2, hsa-miR-107, and hsa-miR-379-5p were found to be known to regulate 625 genes (Supplementary Figure 5C). Furthermore, the miRNAs that were more abundant in EXs including hsa-miR-7704, hsa-miR-200b-3p, hsa-miR-4492, hsa-miR-30c-1-5p, hsa-miR-30c-2-5p and hsa-miR-1273g-3p were found to be known to regulate 595 genes (Supplementary Figure 5D). There were 47 target known to be regulated by miRNAs from both PKC-derived APs and MVs, and PKC derived EXs. Moreover, there were 574 genes and 548 genes that were found to be uniquely regulated by miRNAs from PKC-derived APs and MVs; and PKC-derived EXs, respectively.

Analysis of the biochemical pathways associated with the unique genes regulated by the miRNAs of each EV group using Panther (version 12) showed that the miRNAs derived from APs and MVs that originated from both HaCaT and PKCs regulate genes involved in 14 pathways while the miRNAs derived from EXs that originated from both HaCaT and PKCs regulate genes involved in six pathways (Table 3). The pathways associated with these uniquely regulated genes may indicate some of the particular bioactivities of each EV type.

**Table 3:** Biochemical pathways unique to the group of APs and MVs and the group of EXs from both HaCaT and PKCs.

**Unique pathways to APs and MVs**	**Unique pathways to EXs**
Ornithine degradation	Aminobutyrate degradation
BMP_signaling_pathway-drosophila	mRNA splicing
Adenine and hypoxanthine salvage pathway	Vitamin D metabolism and pathway
DPP_signaling_pathway	Formyltetrahydroformate biosynthesis
GBB_signaling_pathway	Gamma-aminobutyric acid synthesis
ALP23B_signaling_pathway	Nicotine pharmacodynamics pathway
MYO_signaling_pathway	
SCW_signaling_pathway	
Axon guidance mediated by semaphorins	
DPP-SCW_signaling_pathway	
Axon guidance mediated by Slit/Robo	
Xanthine and guanine salvage pathway	
De novo pyrimidine deoxyribonucleotide biosynthesis	
Synaptic_vesicle_trafficking	

## Discussion

The number of studies that have investigated EV biology has substantially increased over the past decade as it has become clear that EVs regulate many biological processes (43). However, one of the challenges of EV studies is characterization of EV subpopulations, which is hindered by the lack of consensus standardized methods of isolation and analysis (8, 44). Differences in biogenesis and physical characteristics such as size, weight, content and buoyancy, overlap between different EV populations, which make them difficult to isolate and characterize. Consequently we employed a range of previously reported methods for this study, including NTA, TEM, confocal microscopy, and immunoblotting, in attempt to harmonize characterization of EV populations by differential centrifugation with filtration. As reported herein, the three EV populations having characteristics consistent with APs, MVs, and EXs, appear to be released by both the HaCaT keratinocyte cell line and PKCs into serum-free culture media.

The challenge of obtaining pure EV sub-fractions is well-described in the literature. For these reasons, it is acknowledged that EV populations cannot be defined based simply on size or buoyant density (8, 42, 44). Our current data reveals that each EV fraction exhibited some characteristics that were indicative of the other fractions. For example, the AP fraction included vesicles <1,000 nm and vesicles that had PS positive/DNA negative signatures which is more indicative of MVs than APs (Figures 1A,C). Similarly, vesicles larger than 150 nm which typically indicate MVs, were also evident in the EX fraction (Figure 1B). These data can thus be interpreted to indicate that: (i) AP preparations contain APs that do not contain fragmented DNA (45), and/or (ii) AP and EX preparations are potentially contaminated with MVs. Importantly, the latter is not without precedent, as others have previously reported that vesicles larger than 150 nm are present in EX fractions (7, 46, 47). Moreover, the larger particles present in the EX fraction (Supplementary Figure 4) may have been due to incomplete EV isolation at the *g* force used in this study (Supplementary Figure 6). Indeed, the size of vesicles can be affected by many factors, including the specific detection technique (46). For example, with regard to TEM, the fixation and staining process can cause some dehydration and shrinkage leading to the collapse of the EXs into the characteristic cup-shape vesicles (36, 46, 48). The shrinkage during staining can also result in the underestimation of the size of vesicles compared to NTA which measure vesicle size by laser light scattering and by tracking Brownian motion of individual vesicles in a liquid (46, 49). Regarding the immunoreactivity data, protein markers may be present in secreted EVs but not in secreting cells. This different distribution of protein markers in secreting cells and secreted EVs potentially indicates selective sorting of proteins into EVs (7, 50, 51). However, it is also possible that protein markers may be present in secreting cells at levels lower than the detection limit of immunoblotting. Furthermore, CD63 was detected in HaCaT-derived EXs only but not in PKC-derived EXs. The diverse enrichment of proteins in EVs has been previously reported as being dependent on the EV type and the cell type from which the EV is released (52, 53). As such, data from this current study are possibly cross-contaminated and this is consistent with previous studies. To eliminate potential cross-contamination, alternative methods have been applied to separate EVs, such as density gradient separation for the isolation of EX, which has limited ability to separate APs from MVs (36, 44). The isolation methods chosen typically depend on the degree of EV purity and EV concentration required by particular studies.

Through utilization of deep sequencing technology, this study has identified large populations of miRNAs in EVs released from cultured HaCaT and freshly isolated human donor PKCs and their respective parental cells. To date, seven published studies have used a deep sequencing approach to profile EV miRNAs (19, 20, 22, 31, 51, 54, 55); however, none has previously utilized this approach to examine the miRNA compliment of keratinocyte-derived EVs. As such, the data presented herein provides valuable information for the future study of EVs in skin. Of particular note, overt differences were evident in EV-derived miRNAs populations between parental cells and their respective EVs (Figures 2A,B, 3). This may indicate the selective sorting of miRNAs into different EV populations during their formation and release process and reflect particular functions of EV populations (11, 56, 57). The selective sorting of miRNAs into EVs might depend on: (i) polyuridylation (a non-template nucleotide) enhanced miRNA incorporation into EVs; (ii) the miRNA maturation pathway that depends on AGO2; (iii) the sequence of mature miRNA; (iv) the abundance levels of complementary 3′ UTR mRNA fragments in EVs; or (v) the binding of miRNAs to heterogeneous nuclear ribonucleoprotein A2B1 (hnRNPA2B1) and subsequent sorting to EVs (58, 59). It is important to note that the mechanism involved in selective miRNA sorting to EVs may be altered given the potential difference in physiological and/or pathological states between donors (59, 60). For example, the level of miR-21 was lower in EX isolated from the healthy donor's serum than those from glioblastoma patients (57). On the other hand, let-7f, miR-20b, and miR-30e-3p were lower in EVs isolated from the plasma of lung carcinoma patients than normal controls (61). In this current study, primary keratinocytes and the HaCaT cell line exhibited potential sorting of unique miRNAs into EVs (Figure 2C). Additionally, the miRNA populations in EVs are also influenced by the source or origin of the secreting cells, number of cell passage, EV isolation methods, and miRNA detection techniques (23, 31, 62–64). miRNAs are highly associated with EV secreting cells, for example members of let-7 family were abundant in EX released from the gastric cancer cell line AZ-P7a but less abundant in EX derived from other cancer cell lines (51). The miR-192 and miR-1207-5p were highly abundant in urine-isolated EX using modified exosome precipitation and much less abundant in those isolated by ultracentrifugation, ultracentrifugation combined to filtration, and ultracentrifugation combined to 30% sucrose cushion (62).

In an attempt to discriminate between the three EV populations, the miRNA content of each was analyzed using Euclidean distance clustering analysis. This analysis indicated that the miRNA profiles of APs and MVs were more similar to each other than to EXs (Figure 5). These results are similar to observations reported in a study of EVs isolated from melanoma cells, where APs and MVs had a greater correlation when compared to EXs (22). It seems that the correlation of miRNA content between EVs depends on both the specific EV population (biogenesis) and also the specific parental cell source (HaCaT cells and individual donors for the PKCs). The latter is supported by evidence from this study which revealed more complex miRNA populations in EVs from freshly isolated PKCs compared to the more homogeneous HaCaT cell line (Figures 5B,D), despite the fact all samples were identically processed. As such, it is quite possible that the variation observed in the miRNA populations from PKC-derived EVs isolated from individual donors may reflect physiological variation between individual donor demographics such as age, diet, sun-exposure or subclinical disease such as diabetes.

Although this current investigation revealed that EVs contain a large number of miRNAs, it is important to determine if those miRNAs have been detected in previous EV studies or are novel. Few studies have investigated EV miRNAs; and to the best of our knowledge no study has investigated keratinocyte-derived EV miRNAs (19, 20, 22, 54). The large number of previously unreported-exosomal miRNAs discovered in this study may be due to the research area being relatively new. Importantly, the ExoCarta database only gathers information for exosomes, leaving a deficit in the curated knowledgebase of AP and MV miRNA cargo. This makes it challenging to interpret the novelty of AP and MV miRNA findings.

Within recent years, consideration of the roles of miRNAs in the regulation of the physiological states of living organisms has increased. Evidence has demonstrated the link between miRNAs and cancer (65, 66), and the connections between miRNAs, including EV miRNAs, with various stages of wound healing biology, such as inflammation, proliferation, angiogenesis and remodeling (reviewed in (67–70)). However, very little practical evidence regarding the association of EV miRNAs and biological events is available. As such, bioinformatic analysis of the genes regulated by EV miRNAs can provide insight as to the relative contribution of EVs and EV miRNAs to various biological activities. For instance, preliminary bioinformatic analysis of genes targeted by miRNAs detected in LIM 1863 colon cancer cell line-derived EVs resulted in various enrichment of biological process, cellular component, and molecular function, such as extracellular matrix, membrane and cancer progression (19). Furthermore, important pathways, such as the p53 signaling pathway; TGF-beta signaling pathway; MAPK signaling pathway; cell cycle; among others, have been associated previously with miRNA regulation (19, 71, 72). In this current study, important and unique pathways were detected for sets of unique target genes regulated by a group of miRNAs associated with both APs and MVs as a group and EXs as a separate group (Table 3). The disparity between the biochemical pathways associated with genes regulated by the miRNAs from each group may arise from the specific differences in the physiological conditions between the different EV types in terms of their biogenesis. Although bioinformatics information may serve to indicate the potential connections between EV miRNAs and functional consequences, it is important that further experiments be performed to more deeply understand the mechanisms of EV miRNA regulation of their target genes and subsequent biological functions.

Taken together, we cannot exclude that minor inconsistencies in sample preparation may be responsible for the observed differences in cellular responses reflecting impurities observed between different donors and EV preparations. However, it is noteworthy that miRNAs in EVs from HaCaTs revealed a clear and consistent separation between EV subpopulations (Figures 5A,C), but greater variations were assayed in miRNA populations in EVs from PKCs (Figures 5B,D). These data suggest that the purity of EV preparations is unlikely to affect cellular responses to EV miRNA populations. The finding that the miRNAs identified within specific EV populations are known to regulate unique sets of target genes associated with particular biochemical pathways support this conclusion. Notwithstanding, a detailed investigation of the mechanistic processes of EV mediated intercellular communication is warranted; however, this was beyond the scope of the study and remains a key area of future investigation.

## Author contributions

UT, DG, DL, JB, CS, and TP contributed in design of the work, analysis and interpretation of data. UT drafted the manuscript. DG, DL, JB, CS, and TP revised the manuscript. All authors have approved the manuscript submission.

### Conflict of interest statement

The authors declare that the research was conducted in the absence of any commercial or financial relationships that could be construed as a potential conflict of interest.
