# Supplementary figures and images for: Differential Expression of Keratinocyte-Derived Extracellular Vesicle Mirnas Discriminate Exosomes From Apoptotic Bodies and Microvesicles
Source: Front Endocrinol (Lausanne). 2018 Sep 11;9:535. doi: 10.3389/fendo.2018.00535 (PMC6143807; doi:10.3389/fendo.2018.00535)

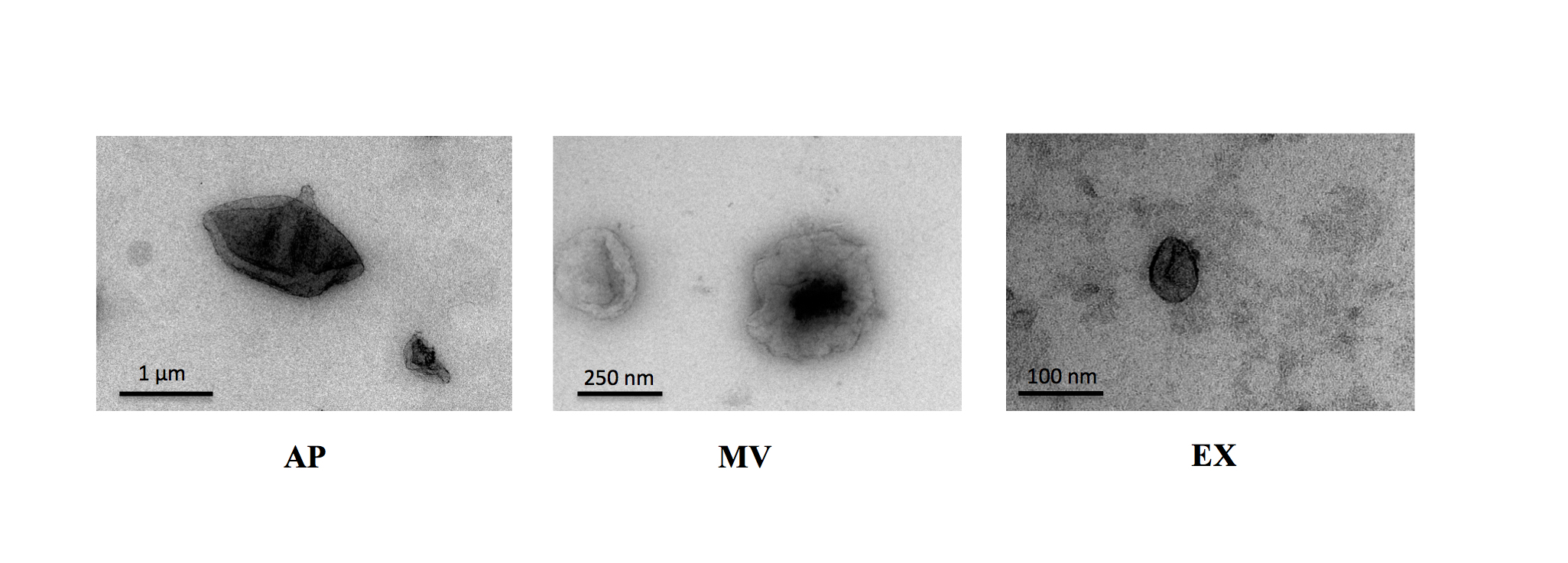

Supplement: Supplementary file 1 [file Image_1.JPEG]

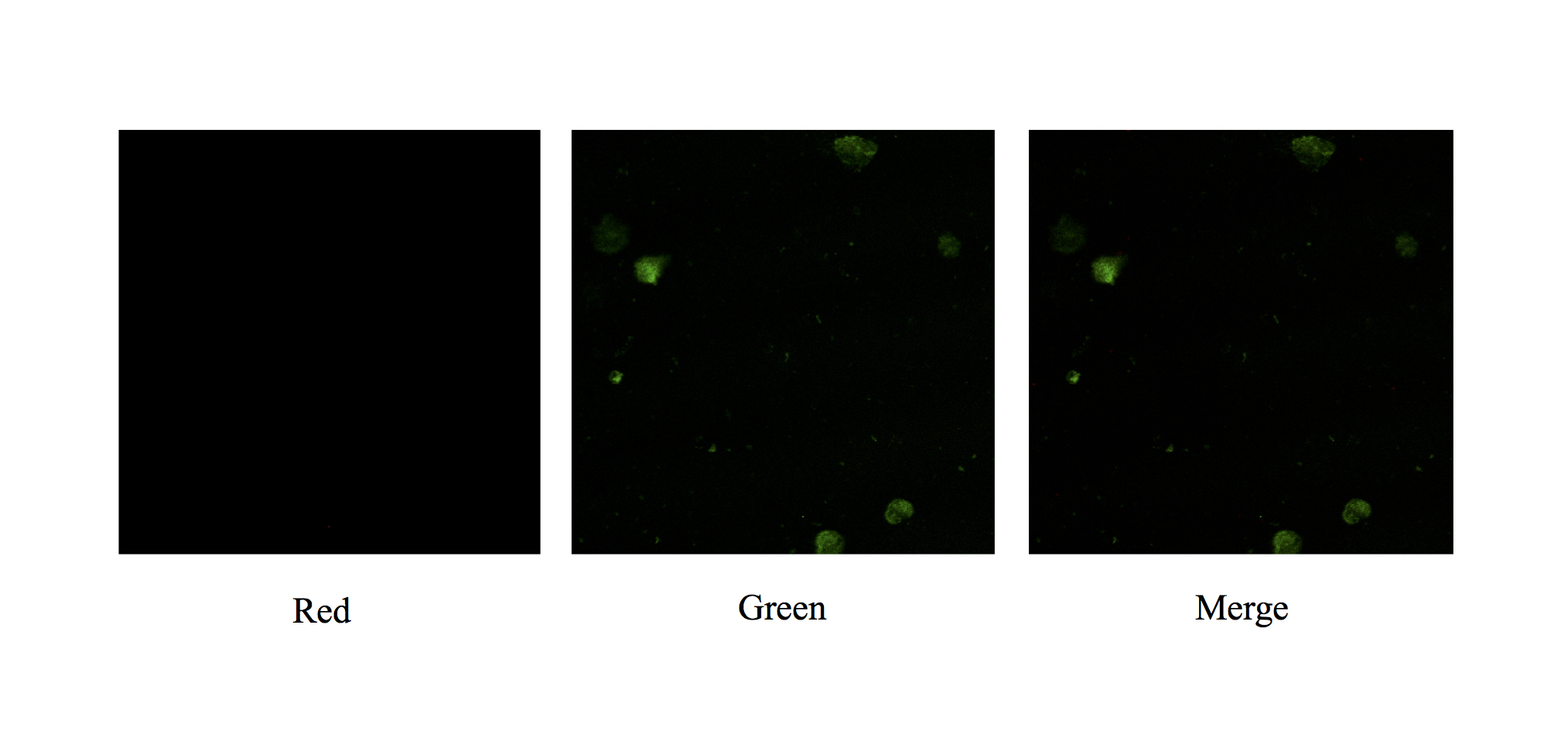

Supplement: Supplementary file 2 [file Image_2.JPEG]

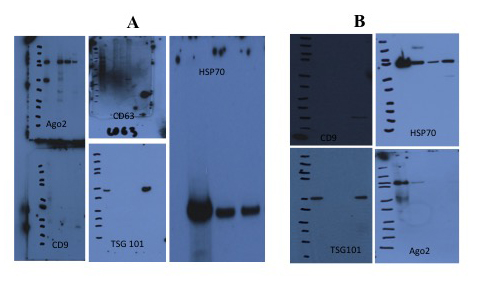

Supplement: Supplementary file 3 [file Image_3.JPEG]

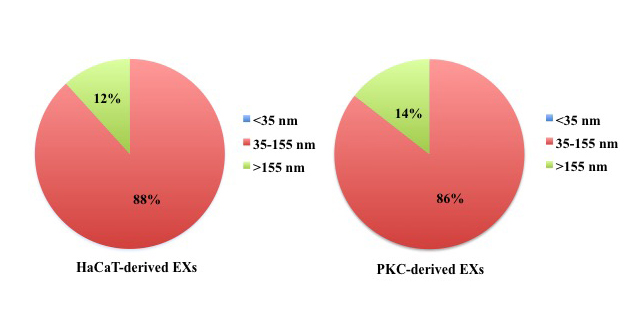

Supplement: Supplementary file 4 [file Image_4.JPEG]

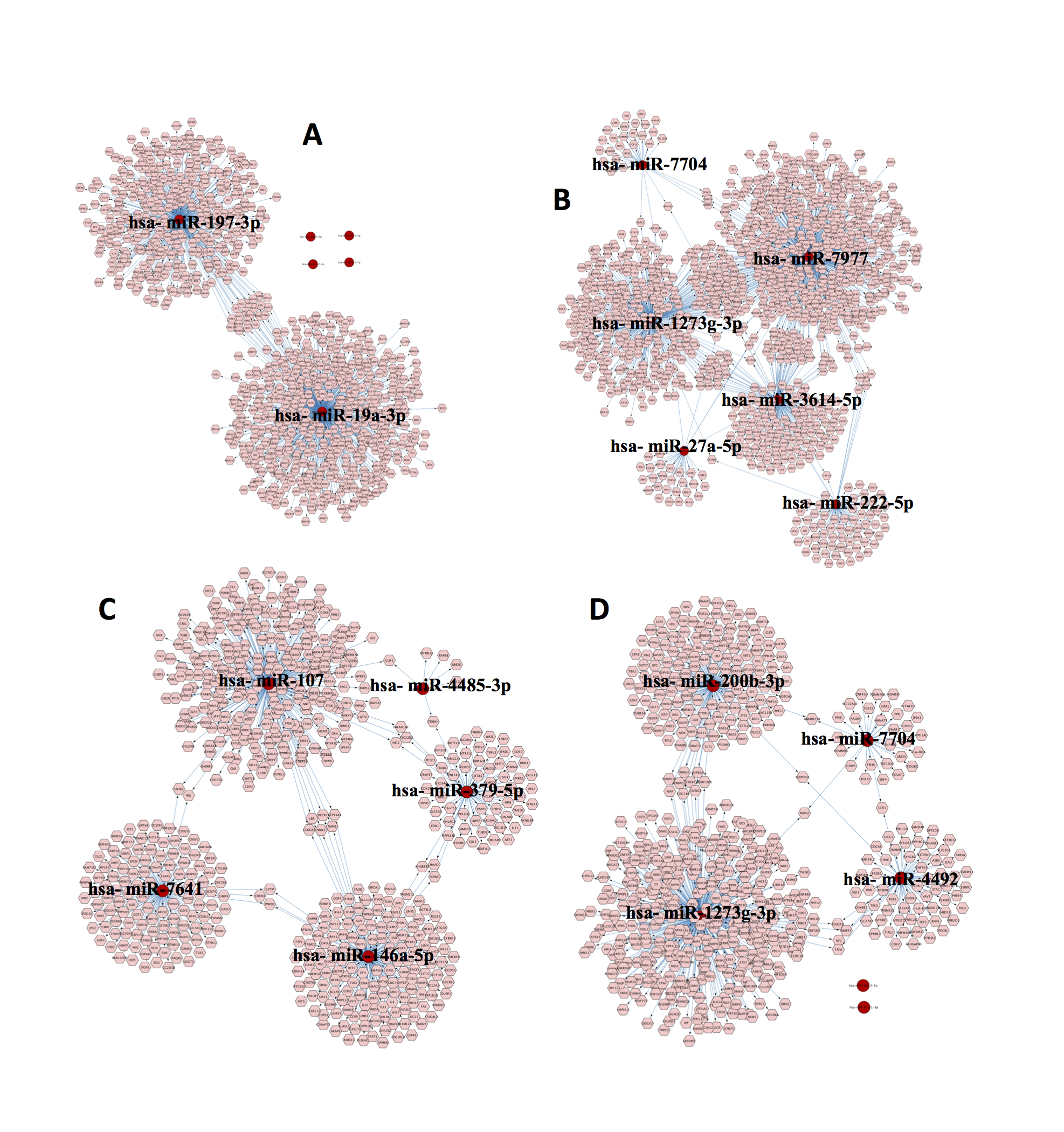

Supplement: Supplementary file 5 [file Image_5.JPEG]

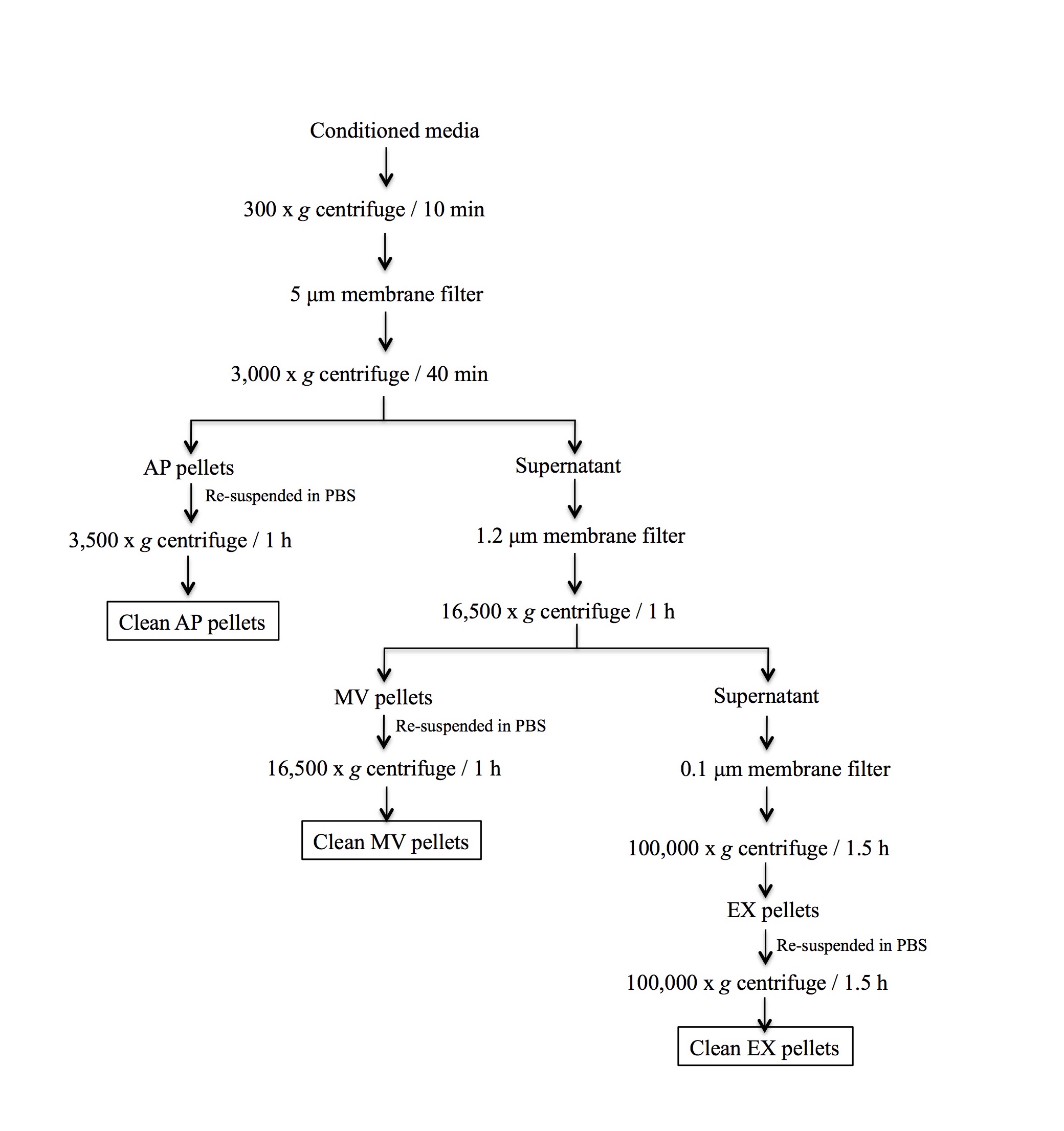

Supplement: Supplementary file 6 [file Image_6.JPEG]

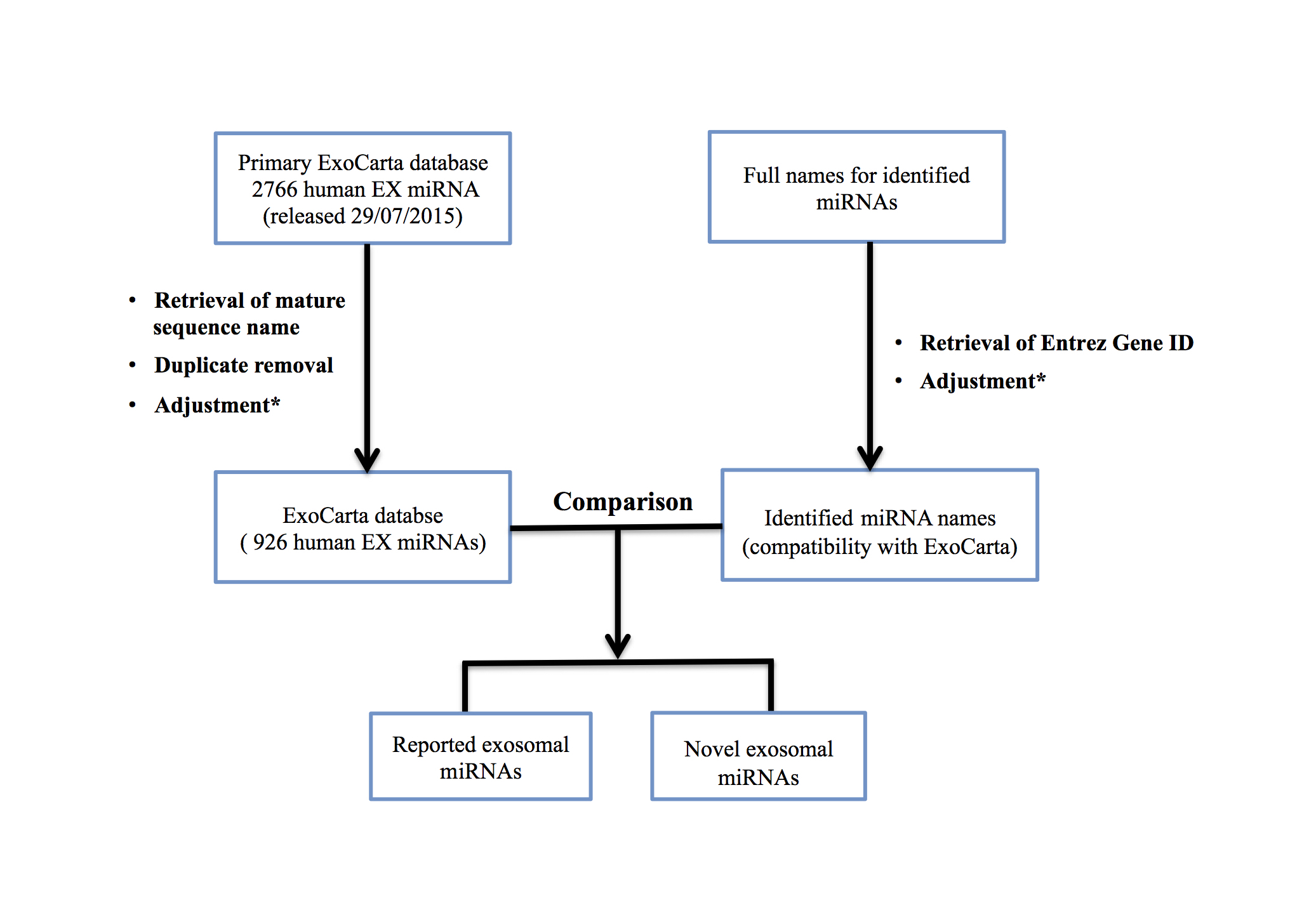

Supplement: Supplementary file 7 [file Image_7.JPEG]
