# Supplementary material for: Differential Expression of Keratinocyte-Derived Extracellular Vesicle Mirnas Discriminate Exosomes From Apoptotic Bodies and Microvesicles
Source: Front Endocrinol (Lausanne). 2018 Sep 11;9:535. doi: 10.3389/fendo.2018.00535 (PMC6143807; doi:10.3389/fendo.2018.00535)
Supplement: Supplementary file 8 [file Data_Sheet_1.DOCX]

Supplementary Material

Differential expression of keratinocyte-derived extracellular vesicle miRNAs discriminate exosomes from apoptotic bodies and microvesicles

Uyen T T Than^1, 2, 6^, Dominic Guanzon^1, 2, 4, 6^, James A Broadbent^2^, David I Leavesley^2,4^, Carlos Salomon^3,5^, and Tony J Parker^1, 2^

**Correspondence:** Uyen Than (uyentpp@yahoo.com), Tony Parker ([a.parker@qut.edu.au](mailto:a.parker@qut.edu.au))

# Supplementary Methods

***Cell culture***

Ethical approval for research detailed herein was obtained from: Queensland University of Technology (QUT); Pacific Day Surgery / Brisbane Private Hospital (approval # 1300000063/QUT); Princess Alexandra Hospital (approval # HREC/06/QPAH/91); Uniting Health Care’s St. Andrews Hospital and Wesley Hospital (approval # 2003/46).

Epidermal primary keratinocytes (PKCs) were freshly isolated from donor skin (surgical discard) and propagated on i3T3 feeder cells (1x10^6^ cells/T75 flask) using the method of Rheinwald and Green [56]: DMEM | Ham’s F12 (3:1), supplemented with 10% foetal calf serum, 2 mM L-glutamine, 1% v/v penicillin-streptomycin, 180 μM adenine, 0.5 μM insulin, 0.05 μM cholera toxin, 0.01% v/v non-essential amino acids solutions, 2.5μg transferrin, 0.1 μM triiodothyronine, 0.16 μg hydrocortisone, 0.05 ng human recombinant EGF [56].

HaCaT cells were purchased from CLS Cell Lines Service GmbH (Eppenheim, Germany) [57]. HaCaT cells seeded at 1-2 x 10^6^ cells/T75 flask were propagated in DMEM supplemented with 10% FCS, 1% pen/strep and 1% glutamine).

All cultures for both PKCs and HaCaT were maintained at 37 ºC in a 5% CO­_2_ / 95% air atmosphere, re-fed every 2 days, and subcultured when cells reach 80% confluency.

***EV production and isolation***

Epidermal primary keratinocytes (passage 2) and HaCaT (passage 49 ≤ 53) were cultured to 80% confluence; the expired media and i3T3 cells were removed and the remaining primary keratinocyte cultures washed twice with fresh, warm, supplement-free DMEM. The cultures were then incubated for 48 hours with serum-free media for EV production. The EV-enriched media released by HaCaT and PKC cultures (CM) were collected and centrifuged at 300 x *g* / 10 minutes to remove cell debris prior to proceeding with EV isolation [58].

EV’s were isolated using a modification of Rossella and Valadi [10, 11]. Briefly, EV enriched CM were gravity filtered through a 5 μm Durapore® membrane filter (Millipore) then centrifuged at 3,000 x *g* for 40 minutes prior to collection and resuspension of the of the AP pellets in PBS, and re-centrifugation at 3,500 x *g* for 1 hour. The resulting pelleted material was designated as clean “AP-harvest”. Supernatants from the AP-harvest were gravity filtered through a 1.2 μm Hydrophilic Nylon membrane filter (Millipore) prior to centrifugation of the filtrates at 16,500 x *g* for 1 hour at 4 ºC (Rotor SW70, Beckman Coulter). The resulting “MV-harvest” pellets were resuspended in PBS and centrifuged at 16,500 x *g* for 1 hour at 4 ºC for clean MV pellet. The supernatant from “MV-harvest” was gravity filtered through a 0.1 μm Durapore® membrane filter (Millipore). The filtrate was then centrifuged at 100,000 x *g* for 1.5 hours at 4 ºC to yield “EX-harvest” pellets. The EX pellets were resuspended in PBS and centrifuged at 100,000 x *g* for 1.5 hours at 4 ºC for clean EXs. Clean AP, MV and EX harvest materials were resuspended in PBS (approximately 30 μl) and stored at -20ºC for up to 4 weeks, or at -80ºC for longer-term storage. The protocol was summarised in Supplemental figure 3.

***Protein extraction***

A volume of AP, MV, EX or cell suspension was admixed with an equal volume of extraction buffer (4 % SDS, 100 mM Tris / HCl pH 7.6) in Protein Lo-Bind tubes and incubated for 3 minutes at 95 ºC. Samples were subsequently sonicated for 5 minutes at room temperature. The mixtures were centrifuged at 14,000 x *g* for 15 minutes at 4 ºC and the protein supernatant was stored at -20 ºC until required. Total protein concentration was determined using the bicinchoninic acid (BCA) protein assay kit (Thermo Scientific™) according to the manufacturer’s instructions.

***Immunobloting***

Cell lysate and EV proteins (20 µg) were separated electrophoretically in 4-12 % SDS-PAGE gels (Invitrogen) at 200 V for 35 minutes. Separated proteins were then electrophoretically transferred onto a pure nitrocellulose membrane (BioTrace™ NT, PALL Life Technology, Mexico) at 200 mA for 2 hours in chilled transfer buffer (25 mM Tris Base, 192 mM Glycine and 20 % (v/v) Methanol). Following transfer, the membrane was blocked with 5 % skim milk in Tris Buffered Saline/ 0.01 % Tween (TBST), pH 7.4, for 30 minutes. Primary antibodies against CD9, CD63, HSP70, and TSG101, and AGO2 (Abcam®) were diluted in 5 % skim milk or albumin in TBST (HSP70: 1/10000; TSG 101: 1/10000; AGO2: 1/5000; CD9: 1/100; and CD63: 1/100). The membrane was then probed overnight at 4 °C with diluted primary antibodies prior to wash and incubation with HRP-conjugated secondary antibody (HAF008, R&D Systems). Antibody binding was detected using the ECL detection solution (Pierce™ ECL Western Blotting Substrate, Thermo Scientific) as per manufacturer’s instructions and imaged on Curix Ultra UV-G Medical X-ray film (AFGA; Mortsel, Belgium).

***Nanoparticle tracking analysis (NTA)***

A volume of 50 µL of EX suspension was diluted with Milli-Q water to a total volume of 500 μL in 1 mL LoBind tubes (Eppendorf). Concentration, size, aggregation and zeta potential of individual EX samples were acquired and analysed using a NanoSight NS500 and Nanoparticle Tracking Analysis (NTA) 3.0 software (Malvern, Worcestershire, UK). A control bead (100 nm) suspension was initially analysed in order to establish the optimal parameters for EX analysis.. Capture parameters were optimised for exosomes; camera level setting = 5; capture number = 5; capture duration = 60 seconds; gain = 1.0; and detection threshold = 10.

***Transmission electron microscopy (TEM) and Confocal microscopy***

EV samples were fixed with saline buffered 4% paraformaldehyde, and deposited onto Formvar-carbon coated grids (Ted Pella, Inc., Redding CA). EV samples were washed 8 times with PBS, negatively stained with uranyl-oxalate and over-layered with methylcellulose. Imaging was performed using a JEOL 1400 Transmission Electron Microscope (TEM) (JEOL Ltd., Tokyo, Japan) at 80 kV.

Clean AP pellets were resuspended in 100 µL of 1 X binding buffer (Annexin V-FITC Apoptosis Detection Kit, Abcam, Cambridge, UK) and stained for phosphatidyl serine (Annexin V-FITC) and nucleic acids (propidium iodide) as per the manufacturer’s instructions. Approximately 5 µL of the AP suspension was applied to a glass slide, observed and photographed (60X objective) using a dual filter set for FITC and rhodamine with a Leica TSC SP5 (Leica Microsystems, Germany).

***Total RNA extraction***

Total RNA was extracted using the Trizol™ method following the manufacturer’s protocol [37, 59]. Briefly, Trizol™ reagent (Thermo Fisher Scientific) was added to 2 mL Eppendorf tubes each containing either whole cell suspension, APs, MVs or EXs in a volume ratio of 9 : 1 (900 µL Trizol : 100 µL cells/vesicles). The Trizol-vesicle solution was triturated or vortexed to ensure vesicle lysis prior to addition of MgCl_2_ solution (Sigma) to a final concentration of 50 mM in order to stabilize RNA structures. For better precipitation yield, Glycogen Azure (Sigma) was added to each mixture to produce a final concentration of no more than 8 μg of Glycogen Azure/ mL in the final RNA solution prior to the addition of 180 μL of chloroform to each sample per 900 µL of Trizol added. Each mixture was vortexed vigorously for 10 seconds, incubated at room temperature for 10 minutes and centrifuged at 12,000 x *g* for 5 minutes prior to the transfer of the aqueous phase to fresh 2 mL eppendorf tubes. Then, 0.9 mL of isopropanol was added per mL of Trizol was added and the samples were inverted 30 times each, incubated at RT for 10 minutes and then incubated at -20 °C for 1 hour (or overnight). Following incubation, the samples were centrifuged at 12,000 x *g* for 10 minutes at 4 °C. The resulting supernatants were carefully aspirated prior to the addition of 1 mL of RNAse-free 75 % ethanol to wash each sample. The samples containing the dislodged pellets were centrifuged at 7,500 x *g* for 5 minutes at 4 °C, washed again with RNAse-free 75 % ethanol prior to removal of the supernatants and allowing the RNA pellets to air dry. Finally, the RNA was resuspended in 10 – 20 μL RNase-free water (Invitrogen) (depending on the size of the RNA pellets).

Total RNA concentration was determined at A260 following application of 1.5 µL of RNA suspension onto a Nanodrop® ND-1000 (Thermo Scientific) spectrometer. The A260 / A280 ratio was used to indicate the purity of samples with a ratio of above 1.8 being considered suitably pure.

After vesicle-derived RNA had been quantified and evaluated for quality, RNA samples of appropriate quality were subjected to initial qRT-PCR experiments to confirm the presence of miRNAs prior to subjecting RNA to next generation sequencing.

***Sequencing microRNAs using Illumina ® Next Seq500***

A RNA library was prepared using the Illumina® TruSeq® Small RNA Library Prep Kit as per the manufacturer’s instructions. Initially, 1 µL of RNA 3’ adapters were mixed with 5 µL of total RNA while on ice, prior to incubation at 70 ºC for 2 minutes and then immediately returned to ice. To start the ligation reaction, 4 µL of reagent mix (2 µL Ligation Buffer, 1 µL RNase Inhibitor and 1 µL T4 RNA Ligase 2) was added into the reaction tube prior to incubation in a thermal cycler (BIO-RAD T100^TM^ Thermal Cycler) at 28 ºC for 1 hour. Next, 1 µL of stop solution was added to the reaction tube prior to incubation at 28 ºC for a further 15 minutes. The reaction was subsequently placed on ice prior to the addition of 3 µL of RNA 5’ adapter mix (1 µL RNA 5’ adapter, 1 µL 10mM ATP and 1 µL T4 RNA Ligase), gentle mixing by trituration, incubation in a thermal cycler at 28 ºC for 1 hour and placement on ice. Reverse transcription was conducted by the addition of 1 µL of RNA RT primer to each 6 µL of 3’ and 5’ adapter-ligated total RNA prior to incubation at 70 ºC for 2 minutes. Next, 5.5 µL of reagent mix (2 µL 5X First Strand Buffer, 0.5 µL 12.5 mM dNTP, 1 µL 100 mM DTT, 1 µL RNase Inhibitor and 1 µL SuperScript II Reverse Transcriptase) was added and incubated at 50 ºC for 1 hour to facilitate the reverse transcription reaction. The resulting cDNA was amplified by PCR using primers designed to anneal to the ends of the adapters. A 37.5 µL volume of PCR master mix (8.5 µL Ultra Pure Water, 25 µL PCR Mix, 2 µL RNA PCR Primer and 2 µL RNA PCR Primer Index) was added to the reaction tube and the PCR conditions were set as follows: thermal cycler lid was preheated to 100 ºC; then the block was heated to 98 ºC for 30 seconds; 11 cycles of 98 ºC for 10 seconds, 60 ºC for 30 seconds and 72 ºC for 15 seconds; 72 ºC for 10 minutes; and holding stage at 4 ºC. The amplified PCR products from this stage were referred to as the small RNA library which was subsequently purified by gel electrophoresis. Briefly, a maximum of 50 µL of the small RNA library was mixed with 10 µL of Novex® Hi-Density TBE Sample Buffer then loaded into two lanes of a Novex 6 % TBE 10-well gel, flanked by custom RNA ladder (CRL) and high-resolution DNA ladder (HRL) (supplied with the Illumina TruSeq small RNA prep kit). The gel was run at 145 V for 60 minutes prior to staining with SYBR gold solution (1X concentration in 50 mL TBE running buffer) and subsequent visualisation in a UV transilluminator. The bands containing miRNAs between 145 bp and 160 bp were excised using a gel breaker tube and collected into a 1 mL LoBind eppendorf tube. The small RNA library was eluted in 200 µL pure water by incubation overnight with shaking and then validated using a Bioanalyzer. The resulting cDNA library was diluted to 2 nM using a solution of Tris – HCl 10 nM, pH 8.5 and 0.1 % Tween 20 prior to loading onto an Illumina chip (75 cycles High Output flow cell) and sequenced using an Illumina® NextSeq500.

***miRNA identification and statistics***

Sequencing using the Illumina® Next Seq500 results in a FASTQ file. Index and adaptor sequences were then removed using the TagCleaner program (http://tagcleaner.sourceforge.net/index.html, version 0.16) and trimmed to 28 nucleotides using the FASTX-Toolkit program (http://hannonlab.cshl.edu/fastx_toolkit/index.html, version 0.0.13) prior to submission of the cleaned nucleotide data to the miRDeep2 software for subsequent analysis. The databases required for this analysis included the human genome (hg19) indexed by Bowtie (downloaded from http://bowtie-bio.sourceforge.net/index.shtml) and miRNA databases “mature.fa” and “hairpin.fa” (downloaded from <http://www.mirbase.org/index.shtml>). The human mature and hairpin miRNAs (from mature.fa and hairpin.fa databases) were extracted and the sequences were aligned to the humane genome (hg19) using the mapper module in miRDeep2. Next the quantifier module in miRDeep2 was used to quantify miRNAs and finally generate a file which contained a summary of identified and quantified miRNAs for each sample.

The identified miRNAs and their raw counts were further analysed using the DESeq2 package (version 1.10.1) for filtering, normalisation and to test the differential expression of miRNA levels using a negative binomial generalised linear model [60]. A Wald test was used to calculate statistical significance and was adjusted for multiple testing using the Benjamini and Hochberg procedure [60]. Results were considered statistically different where there was an adjusted p-value < 0.01 between groups. Graphs and heatmaps were produced using the R statistical environment (R version 3.2.2, last update 14/8/2015) and gplots package (version 2.17.0) [61].

***ExoCarta database***

To determine if any of the detected exosomal miRNAs were unique and had not been previously reported, the lists of common and exosomal miRNAs were compared with the ExoCarta miRNA database. The miRNA database was downloaded from the ExoCarta website (exocarta.org, version 5, released on 29 July 2015) which contained a total of 2766 miRNAs (*Homo sapiens*). Since not all of the miRNA names in the database contain the stem loop component of the full miRNA name, these were added manually following retrieval from the miRTarbase website through matching of the Entrez Gene ID with the miRNA name information. Next, the annotated miRNA list was sorted to remove duplicates (miRNAs detected by different methods) followed by removal of the lettered suffixes which indicate 5’ arm or 3’ arm of the precursor miRNAs in order to retrieve miRNA names for only mature sequences. This produced a final list of 926 miRNAs for the comparison. The empirical exosomal miRNA lists (with at least two counts) were used to search the DAVID database to obtain the Entrez Gene ID for each identified miRNA (<https://david.ncifcrf.gov/>). Similarly, exosomal miRNA names were then adjusted to the Entrez Gene ID to ensure compatibility with the ExoCarta database. A summary of the procedure is illustrated in Supplemental figure 4.

# Supplementary Figures and Tables

## Supplementary Figures

Supplementary figure 1: Representatives of three EV sub-populations released from primary keratinocytes. AP: Apoptotic body; MV: Microvesicle; EX: Exosome.

Supplementary figure 2: MV fractions were stained with PI (red) and AnnexinV (green) to reveal the presence of nucleic acid fragments and expression of PS - Annexin V respectively. Staining with PI and Annexin V revealed MVs were negative for nuclear fragments but positive for Annexin V. Images are representative for MVs released from primary keratinocytes (isolated from donor # 288.) The stained MVs were examined and photographed using Leica TSC SP5 Confocal microscopy, objective 60X.

Supplementary figure 3: Original images of EV markers reported in the main text. A) Images of protein markers from HaCaT cell line and HaCaT-derived EVs. B) Images of protein markers from primary keratinocytes and primary keratinocyte-derived EVs.

Supplementary figure 4: Percentage of EV distribution in EX preparation.

Supplementary figure 5: Analysis of target genes regulated by miRNAs associated with select APs and MVs and EXs using Cytoscape. A) Network of target genes regulated by miRNAs more abundant in HaCaT-derived APs and MVs. B) Network of target genes regulated by miRNAs more abundant in HaCaT-derived EXs. C) Network of target genes regulated by miRNAs more abundant in PKC-derived APs and MVs. A) Network of target genes regulated by miRNAs more abundant in PKC-derived EXs.

Supplementary figure 6: EV isolation steps from keratinocyte culture media.

Supplementary figure 7: Summary of the EX miRNA refining procedure. (*) A list of EX miRNAs downloaded from ExoCarta database was refined to select EX miRNAs from homosapiens and adjusted by manually adding the stem loop component of the full miRNA name. To retrieve miRNA names for only mature sequences duplicates were removed from the annotated miRNA list followed by removal of the lettered suffixes which indicate the 5’ arm or 3’ arm of the precursor miRNAs. Similarly, exosomal miRNA names detected in this current study were then adjusted to the Entrez Gene ID to ensure compatibility with the ExoCarta database.

## Supplementary tables

Supplementary table 1: List of 381 shared miRNAs between common miRNAs from HaCaT- and primary keratinocyte-derived EVs

| ***miRNA name*** | | | |
| --- | --- | --- | --- |
| hsa-let-7a-1-3p | hsa-mir-16-2-3p | hsa-mir-29b-2-3p | hsa-mir-4520-1-3p |
| hsa-let-7a-1-5p | hsa-mir-16-2-5p | hsa-mir-29c-3p | hsa-mir-4520-2-3p |
| hsa-let-7a-2-5p | hsa-mir-17-3p | hsa-mir-29c-5p | hsa-mir-454-3p |
| hsa-let-7a-3-3p | hsa-mir-17-5p | hsa-mir-301a-3p | hsa-mir-454-5p |
| hsa-let-7a-3-5p | hsa-mir-181a-1-3p | hsa-mir-301b-3p | hsa-mir-455-3p |
| hsa-let-7b-3p | hsa-mir-181a-1-5p | hsa-mir-3065-3p | hsa-mir-455-5p |
| hsa-let-7b-5p | hsa-mir-181a-2-3p | hsa-mir-3065-5p | hsa-mir-4775 |
| hsa-let-7c-5p | hsa-mir-181a-2-5p | hsa-mir-30a-3p | hsa-mir-4792 |
| hsa-let-7d-3p | hsa-mir-181b-1-5p | hsa-mir-30a-5p | hsa-mir-484 |
| hsa-let-7d-5p | hsa-mir-181b-2-5p | hsa-mir-30b-5p | hsa-mir-486-1-5p |
| hsa-let-7e-3p | hsa-mir-181c-3p | hsa-mir-30c-1-3p | hsa-mir-486-2-5p |
| hsa-let-7e-5p | hsa-mir-181c-5p | hsa-mir-30c-1-5p | hsa-mir-487b-3p |
| hsa-let-7f-1-3p | hsa-mir-181d-5p | hsa-mir-30c-2-3p | hsa-mir-493-3p |
| hsa-let-7f-1-5p | hsa-mir-182-5p | hsa-mir-30c-2-5p | hsa-mir-493-5p |
| hsa-let-7f-2-5p | hsa-mir-183-3p | hsa-mir-30d-3p | hsa-mir-497-5p |
| hsa-let-7g-5p | hsa-mir-183-5p | hsa-mir-30d-5p | hsa-mir-500a-3p |
| hsa-let-7i-3p | hsa-mir-184 | hsa-mir-30e-3p | hsa-mir-501-3p |
| hsa-let-7i-5p | hsa-mir-185-5p | hsa-mir-30e-5p | hsa-mir-502-3p |
| hsa-mir-100-3p | hsa-mir-186-5p | hsa-mir-31-3p | hsa-mir-505-3p |
| hsa-mir-100-5p | hsa-mir-188-5p | hsa-mir-31-5p | hsa-mir-509-1-3p |
| hsa-mir-101-1-3p | hsa-mir-18a-3p | hsa-mir-3158-1-3p | hsa-mir-509-2-3p |
| hsa-mir-101-2-3p | hsa-mir-18a-5p | hsa-mir-3158-2-3p | hsa-mir-509-3-3p |
| hsa-mir-103a-1-3p | hsa-mir-190a-5p | hsa-mir-3176 | hsa-mir-5096 |
| hsa-mir-103a-2-3p | hsa-mir-191-5p | hsa-mir-3178 | hsa-mir-5100 |
| hsa-mir-106b-3p | hsa-mir-1910-5p | hsa-mir-3195 | hsa-mir-532-3p |
| hsa-mir-106b-5p | hsa-mir-192-5p | hsa-mir-3196 | hsa-mir-532-5p |
| hsa-mir-107 | hsa-mir-193a-3p | hsa-mir-32-5p | hsa-mir-542-3p |
| hsa-mir-10a-5p | hsa-mir-193b-3p | hsa-mir-320a | hsa-mir-548e-3p |
| hsa-mir-10b-5p | hsa-mir-193b-5p | hsa-mir-320b-1 | hsa-mir-548k |
| hsa-mir-1180-3p | hsa-mir-194-1-5p | hsa-mir-320b-2 | hsa-mir-548o-2-3p |
| hsa-mir-1246 | hsa-mir-194-2-5p | hsa-mir-320c-1 | hsa-mir-548o-3p |
| hsa-mir-125a-3p | hsa-mir-195-5p | hsa-mir-320c-2 | hsa-mir-550a-1-3p |
| hsa-mir-125a-5p | hsa-mir-196a-1-5p | hsa-mir-324-3p | hsa-mir-550a-2-3p |
| hsa-mir-125b-1-3p | hsa-mir-196a-2-5p | hsa-mir-324-5p | hsa-mir-550a-3-3p |
| hsa-mir-125b-1-5p | hsa-mir-196b-5p | hsa-mir-328-3p | hsa-mir-5585-3p |
| hsa-mir-125b-2-3p | hsa-mir-197-3p | hsa-mir-330-3p | hsa-mir-561-5p |
| hsa-mir-125b-2-5p | hsa-mir-199a-1-3p | hsa-mir-330-5p | hsa-mir-574-3p |
| hsa-mir-126-3p | hsa-mir-199a-1-5p | hsa-mir-331-3p | hsa-mir-574-5p |
| hsa-mir-126-5p | hsa-mir-199a-2-3p | hsa-mir-331-5p | hsa-mir-576-5p |
| hsa-mir-1260a | hsa-mir-199a-2-5p | hsa-mir-335-5p | hsa-mir-582-3p |
| hsa-mir-1260b | hsa-mir-199b-3p | hsa-mir-339-3p | hsa-mir-582-5p |
| hsa-mir-1268a | hsa-mir-199b-5p | hsa-mir-339-5p | hsa-mir-584-5p |
| hsa-mir-1268b | hsa-mir-19a-3p | hsa-mir-33a-5p | hsa-mir-589-3p |
| hsa-mir-127-3p | hsa-mir-19b-1-3p | hsa-mir-33b-5p | hsa-mir-589-5p |
| hsa-mir-1271-5p | hsa-mir-19b-2-3p | hsa-mir-340-5p | hsa-mir-590-3p |
| hsa-mir-1273g-3p | hsa-mir-200a-3p | hsa-mir-342-3p | hsa-mir-598-3p |
| hsa-mir-1275 | hsa-mir-200a-5p | hsa-mir-345-5p | hsa-mir-6087 |
| hsa-mir-1277-5p | hsa-mir-200b-3p | hsa-mir-34a-5p | hsa-mir-615-3p |
| hsa-mir-128-1-3p | hsa-mir-200b-5p | hsa-mir-34c-5p | hsa-mir-619-5p |
| hsa-mir-128-2-3p | hsa-mir-200c-3p | hsa-mir-361-3p | hsa-mir-627-3p |
| hsa-mir-1285-1-3p | hsa-mir-203a-3p | hsa-mir-361-5p | hsa-mir-629-5p |
| hsa-mir-1285-1-5p | hsa-mir-203a-5p | hsa-mir-3614-5p | hsa-mir-641 |
| hsa-mir-1285-2-3p | hsa-mir-203b-3p | hsa-mir-3615 | hsa-mir-6499-5p |
| hsa-mir-1290 | hsa-mir-205-3p | hsa-mir-362-5p | hsa-mir-651-5p |
| hsa-mir-1293 | hsa-mir-205-5p | hsa-mir-3656 | hsa-mir-6510-3p |
| hsa-mir-1296-5p | hsa-mir-20a-5p | hsa-mir-365a-3p | hsa-mir-652-3p |
| hsa-mir-1301-3p | hsa-mir-21-3p | hsa-mir-365b-3p | hsa-mir-654-3p |
| hsa-mir-1304-3p | hsa-mir-21-5p | hsa-mir-369-3p | hsa-mir-660-5p |
| hsa-mir-1307-3p | hsa-mir-210-3p | hsa-mir-374a-3p | hsa-mir-664a-3p |
| hsa-mir-1307-5p | hsa-mir-210-5p | hsa-mir-374a-5p | hsa-mir-665 |
| hsa-mir-130a-3p | hsa-mir-2110 | hsa-mir-374b-5p | hsa-mir-671-3p |
| hsa-mir-130b-3p | hsa-mir-2116-3p | hsa-mir-375 | hsa-mir-7-1-3p |
| hsa-mir-132-3p | hsa-mir-212-3p | hsa-mir-376a-1-5p | hsa-mir-708-3p |
| hsa-mir-132-5p | hsa-mir-215-5p | hsa-mir-378a-3p | hsa-mir-708-5p |
| hsa-mir-135b-3p | hsa-mir-218-1-5p | hsa-mir-378c | hsa-mir-744-5p |
| hsa-mir-135b-5p | hsa-mir-218-2-5p | hsa-mir-378d-1 | hsa-mir-7641-1 |
| hsa-mir-136-3p | hsa-mir-22-3p | hsa-mir-378d-2 | hsa-mir-7641-2 |
| hsa-mir-138-1-3p | hsa-mir-22-5p | hsa-mir-378e | hsa-mir-769-5p |
| hsa-mir-138-1-5p | hsa-mir-221-3p | hsa-mir-378i | hsa-mir-7704 |
| hsa-mir-138-2-5p | hsa-mir-221-5p | hsa-mir-381-3p | hsa-mir-7706 |
| hsa-mir-140-3p | hsa-mir-222-3p | hsa-mir-3909 | hsa-mir-7977 |
| hsa-mir-140-5p | hsa-mir-222-5p | hsa-mir-3960 | hsa-mir-873-5p |
| hsa-mir-141-3p | hsa-mir-224-3p | hsa-mir-409-3p | hsa-mir-874-3p |
| hsa-mir-141-5p | hsa-mir-224-5p | hsa-mir-410-3p | hsa-mir-877-5p |
| hsa-mir-142-5p | hsa-mir-2355-5p | hsa-mir-411-5p | hsa-mir-92a-1-3p |
| hsa-mir-143-3p | hsa-mir-23a-3p | hsa-mir-421 | hsa-mir-92a-2-3p |
| hsa-mir-145-3p | hsa-mir-23b-3p | hsa-mir-423-3p | hsa-mir-92b-3p |
| hsa-mir-145-5p | hsa-mir-24-1-3p | hsa-mir-423-5p | hsa-mir-93-3p |
| hsa-mir-146a-5p | hsa-mir-24-2-3p | hsa-mir-424-3p | hsa-mir-93-5p |
| hsa-mir-146b-5p | hsa-mir-24-2-5p | hsa-mir-424-5p | hsa-mir-941-1 |
| hsa-mir-147b | hsa-mir-25-3p | hsa-mir-425-3p | hsa-mir-941-2 |
| hsa-mir-148a-3p | hsa-mir-25-5p | hsa-mir-425-5p | hsa-mir-941-3 |
| hsa-mir-148a-5p | hsa-mir-26a-1-5p | hsa-mir-4286 | hsa-mir-941-4 |
| hsa-mir-148b-3p | hsa-mir-26a-2-5p | hsa-mir-429 | hsa-mir-941-5 |
| hsa-mir-148b-5p | hsa-mir-26b-3p | hsa-mir-431-5p | hsa-mir-942-5p |
| hsa-mir-149-5p | hsa-mir-26b-5p | hsa-mir-432-5p | hsa-mir-944 |
| hsa-mir-151a-3p | hsa-mir-27a-3p | hsa-mir-4448 | hsa-mir-95-3p |
| hsa-mir-151a-5p | hsa-mir-27a-5p | hsa-mir-4454 | hsa-mir-96-5p |
| hsa-mir-151b | hsa-mir-27b-3p | hsa-mir-4485-3p | hsa-mir-98-3p |
| hsa-mir-152-3p | hsa-mir-27b-5p | hsa-mir-4492 | hsa-mir-98-5p |
| hsa-mir-152-5p | hsa-mir-28-3p | hsa-mir-4508 | hsa-mir-99a-5p |
| hsa-mir-155-5p | hsa-mir-28-5p | hsa-mir-450a-1-5p | hsa-mir-99b-3p |
| hsa-mir-15a-5p | hsa-mir-296-3p | hsa-mir-450a-2-5p | hsa-mir-99b-5p |
| hsa-mir-15b-3p | hsa-mir-29a-3p | hsa-mir-450b-5p |  |
| hsa-mir-15b-5p | hsa-mir-29a-5p | hsa-mir-4516 |  |
| hsa-mir-16-1-5p | hsa-mir-29b-1-3p | hsa-mir-452-5p |  |

Supplementary table 2: List of exosomal miRNAs from HaCaT- and PKC- derived EXs reported the first time herein

| ***Novel miRNA from HaCaT-derived EXs*** | | | |
| --- | --- | --- | --- |
| hsa-mir-1226-3p | hsa-mir-3942-5p | hsa-mir-514a-3-3p | hsa-mir-6501-5p |
| hsa-mir-1244-2 | hsa-mir-425-3p | hsa-mir-548aa-1 | hsa-mir-651-5p |
| hsa-mir-1244-3 | hsa-mir-425-5p | hsa-mir-548aa-2 | hsa-mir-6510-3p |
| hsa-mir-1244-4 | hsa-mir-4301 | hsa-mir-548ab | hsa-mir-6511a-1-3p |
| hsa-mir-1253 | hsa-mir-4435-1 | hsa-mir-548ae-2-5p | hsa-mir-6511a-2-3p |
| hsa-mir-1254-1 | hsa-mir-4435-2 | hsa-mir-548aj-2-5p | hsa-mir-6511a-3-3p |
| hsa-mir-1254-2 | hsa-mir-4444-1 | hsa-mir-548am-3p | hsa-mir-6511a-4-3p |
| hsa-mir-1255a | hsa-mir-4444-2 | hsa-mir-548am-5p | hsa-mir-6513-3p |
| hsa-mir-1260a | hsa-mir-4449 | hsa-mir-548aq-3p | hsa-mir-6516-3p |
| hsa-mir-1273a | hsa-mir-4459 | hsa-mir-548ar-5p | hsa-mir-663a |
| hsa-mir-1273d | hsa-mir-4477b | hsa-mir-548au-5p | hsa-mir-6716-3p |
| hsa-mir-1273e | hsa-mir-4479 | hsa-mir-548av-3p | hsa-mir-6719-3p |
| hsa-mir-1273f | hsa-mir-4485-3p | hsa-mir-548ay-5p | hsa-mir-6720-3p |
| hsa-mir-1286 | hsa-mir-449b-5p | hsa-mir-548d-1-5p | hsa-mir-6720-5p |
| hsa-mir-129 | hsa-mir-449c-5p | hsa-mir-548d-2-5p | hsa-mir-6723-5p |
| hsa-mir-1296-3p | hsa-mir-4504 | hsa-mir-548e-3p | hsa-mir-6724-1-5p |
| hsa-mir-1303 | hsa-mir-4505 | hsa-mir-548e-5p | hsa-mir-6724-2-5p |
| hsa-mir-1343-3p | hsa-mir-450a-2-5p | hsa-mir-548f-1-3p | hsa-mir-6724-3-5p |
| hsa-mir-1469 | hsa-mir-4511 | hsa-mir-548f-1-5p | hsa-mir-6724-4-5p |
| hsa-mir-1908-3p | hsa-mir-4516 | hsa-mir-548f-2-3p | hsa-mir-6735-5p |
| hsa-mir-23c | hsa-mir-4520-1-3p | hsa-mir-548f-3-3p | hsa-mir-6737-3p |
| hsa-mir-301b-3p | hsa-mir-4520-2-3p | hsa-mir-548f-4-3p | hsa-mir-6754-3p |
| hsa-mir-3065-3p | hsa-mir-4633-3p | hsa-mir-548g-5p | hsa-mir-6767-5p |
| hsa-mir-3127-5p | hsa-mir-4636 | hsa-mir-548h-1-5p | hsa-mir-6777-5p |
| hsa-mir-3130-1-3p | hsa-mir-4639-5p | hsa-mir-548h-2-5p | hsa-mir-6796-5p |
| hsa-mir-3130-2-3p | hsa-mir-4661-5p | hsa-mir-548h-3-5p | hsa-mir-6807-5p |
| hsa-mir-3142 | hsa-mir-4666a-5p | hsa-mir-548h-4-3p | hsa-mir-6824-3p |
| hsa-mir-3142_var2 | hsa-mir-4676-3p | hsa-mir-548h-4-5p | hsa-mir-6829-5p |
| hsa-mir-3157-3p | hsa-mir-4677-3p | hsa-mir-548h-5-5p | hsa-mir-6836-5p |
| hsa-mir-3158-1-3p | hsa-mir-4677-5p | hsa-mir-548o-2-3p | hsa-mir-6869-5p |
| hsa-mir-3158-2-3p | hsa-mir-4686 | hsa-mir-548o-2-5p | hsa-mir-6894-5p |
| hsa-mir-3159 | hsa-mir-4691-5p | hsa-mir-548p | hsa-mir-7-1-5p |
| hsa-mir-3160-1-3p | hsa-mir-4697-3p | hsa-mir-548x-5p | hsa-mir-7-2-5p |
| hsa-mir-3160-2-3p | hsa-mir-4709-3p | hsa-mir-550 | hsa-mir-7-3-5p |
| hsa-mir-3166 | hsa-mir-4709-5p | hsa-mir-556-3p | hsa-mir-7161-5p |
| hsa-mir-3178 | hsa-mir-4714-3p | hsa-mir-556-5p | hsa-mir-7641-1 |
| hsa-mir-3195 | hsa-mir-4717-3p | hsa-mir-5579-3p | hsa-mir-7641-2 |
| hsa-mir-3196 | hsa-mir-4738-3p | hsa-mir-5585-3p | hsa-mir-7703 |
| hsa-mir-320e | hsa-mir-4741 | hsa-mir-561-5p | hsa-mir-7847-3p |
| hsa-mir-3651 | hsa-mir-4746-5p | hsa-mir-5699-3p | hsa-mir-7975 |
| hsa-mir-3684 | hsa-mir-4755-3p | hsa-mir-5700 | hsa-mir-7976 |
| hsa-mir-3687-1 | hsa-mir-4775 | hsa-mir-5701-1 | hsa-mir-7977 |
| hsa-mir-3687-2 | hsa-mir-4797-3p | hsa-mir-5701-2 | hsa-mir-8061 |
| hsa-mir-376a-1-5p | hsa-mir-4800-3p | hsa-mir-5701-3 | hsa-mir-9 |
| hsa-mir-378d-2 | hsa-mir-486 | hsa-mir-580-3p | hsa-mir-934 |
| hsa-mir-378h | hsa-mir-4999-5p | hsa-mir-598-3p | hsa-mir-937-3p |
| hsa-mir-378i | hsa-mir-509-1-3p | hsa-mir-600 | hsa-mir-937-5p |
| hsa-mir-3912-3p | hsa-mir-509-2-3p | hsa-mir-619-5p | hsa-mir-941-1 |
| hsa-mir-3928-3p | hsa-mir-509-3-3p | hsa-mir-627-3p | hsa-mir-941-2 |
| hsa-mir-3934-5p | hsa-mir-5095 | hsa-mir-627-5p | hsa-mir-941-3 |
| hsa-mir-3935 | hsa-mir-5096 | hsa-mir-636 | hsa-mir-941-4 |
| hsa-mir-3940-3p | hsa-mir-514a-1-3p | hsa-mir-643 | hsa-mir-941-5 |
| hsa-mir-3942-3p | hsa-mir-514a-2-3p | hsa-mir-6499-5p | hsa-mir-944 |
| ***Primary keratinocyte-derived Exs*** | | | |
| hsa-mir-1199-5p | hsa-mir-3648-1 | hsa-mir-4743-3p | hsa-mir-6511a-2-3p |
| hsa-mir-1253 | hsa-mir-3648-2 | hsa-mir-4775 | hsa-mir-6511a-3-3p |
| hsa-mir-1254-1 | hsa-mir-3687-1 | hsa-mir-486 | hsa-mir-6511a-4-3p |
| hsa-mir-1254-2 | hsa-mir-3687-2 | hsa-mir-5001-3p | hsa-mir-6511b-1-5p |
| hsa-mir-1260a | hsa-mir-3688-1-3p | hsa-mir-509-1-3p | hsa-mir-6511b-2-5p |
| hsa-mir-1273d | hsa-mir-3688-2-3p | hsa-mir-509-2-3p | hsa-mir-6513-3p |
| hsa-mir-1273e | hsa-mir-3692-5p | hsa-mir-509-3-3p | hsa-mir-6724-1-5p |
| hsa-mir-1273f | hsa-mir-376a-1-5p | hsa-mir-5095 | hsa-mir-6724-2-5p |
| hsa-mir-1273h-5p | hsa-mir-378d-2 | hsa-mir-5096 | hsa-mir-6724-3-5p |
| hsa-mir-1286 | hsa-mir-378i | hsa-mir-548am-5p | hsa-mir-6724-4-5p |
| hsa-mir-129 | hsa-mir-3912-3p | hsa-mir-548au-5p | hsa-mir-6753-3p |
| hsa-mir-1303 | hsa-mir-3928-3p | hsa-mir-548ba | hsa-mir-6787-3p |
| hsa-mir-134-5p | hsa-mir-3929 | hsa-mir-548e-3p | hsa-mir-6824-3p |
| hsa-mir-1469 | hsa-mir-425-3p | hsa-mir-548e-5p | hsa-mir-6840-5p |
| hsa-mir-1972-1 | hsa-mir-425-5p | hsa-mir-548h-4-3p | hsa-mir-6866-5p |
| hsa-mir-1972-2 | hsa-mir-4435-1 | hsa-mir-548o-2-3p | hsa-mir-6886-5p |
| hsa-mir-301b-3p | hsa-mir-4435-2 | hsa-mir-548o-2-5p | hsa-mir-6887-5p |
| hsa-mir-3065-3p | hsa-mir-4459 | hsa-mir-548u | hsa-mir-7641-1 |
| hsa-mir-3130-1-3p | hsa-mir-4485-3p | hsa-mir-550 | hsa-mir-7641-2 |
| hsa-mir-3130-2-3p | hsa-mir-4505 | hsa-mir-5585-3p | hsa-mir-7703 |
| hsa-mir-3130-2-5p | hsa-mir-450a-2-5p | hsa-mir-561-5p | hsa-mir-7851-3p |
| hsa-mir-3155b | hsa-mir-4512 | hsa-mir-5680 | hsa-mir-7854-3p |
| hsa-mir-3158-1-3p | hsa-mir-4516 | hsa-mir-5700 | hsa-mir-7975 |
| hsa-mir-3158-2-3p | hsa-mir-4520-1-3p | hsa-mir-5701-1 | hsa-mir-7976 |
| hsa-mir-3166 | hsa-mir-4520-2-3p | hsa-mir-5701-2 | hsa-mir-7977 |
| hsa-mir-3178 | hsa-mir-4527 | hsa-mir-5701-3 | hsa-mir-8061 |
| hsa-mir-3180-1 | hsa-mir-4532 | hsa-mir-598-3p | hsa-mir-889-3p |
| hsa-mir-3180-1-3p | hsa-mir-4642 | hsa-mir-6126 | hsa-mir-934 |
| hsa-mir-3180-2 | hsa-mir-4661-3p | hsa-mir-619-5p | hsa-mir-937-3p |
| hsa-mir-3180-2-3p | hsa-mir-4677-3p | hsa-mir-627-3p | hsa-mir-937-5p |
| hsa-mir-3180-3 | hsa-mir-4697-3p | hsa-mir-627-5p | hsa-mir-941-1 |
| hsa-mir-3180-3-3p | hsa-mir-4700-3p | hsa-mir-642a-3p | hsa-mir-941-2 |
| hsa-mir-3180-4 | hsa-mir-4700-5p | hsa-mir-642a-5p | hsa-mir-941-3 |
| hsa-mir-3180-4-3p | hsa-mir-4707-5p | hsa-mir-6499-5p | hsa-mir-941-4 |
| hsa-mir-3180-5 | hsa-mir-4709-3p | hsa-mir-6501-5p | hsa-mir-941-5 |
| hsa-mir-3180-5-3p | hsa-mir-4709-5p | hsa-mir-651-5p | hsa-mir-944 |
| hsa-mir-3195 | hsa-mir-4717-3p | hsa-mir-6510-3p | hsa-mir-6511a-1-3p |
| hsa-mir-3196 | hsa-mir-4741 |  | |

Supplementary table 3: List of differential expression miRNAs in HaCaT and PKC samples

| ***HaCaT sample*** | | | | | | |
| --- | --- | --- | --- | --- | --- | --- |
| **miRNA Name** | | **p-adjust** | | **miRNA Name** | | **p-adjust** |
| hsa-miR-222-5p | | 3.31E-29 | | hsa-miR-486-2-3p | | 0.001597449 |
| hsa-miR-1273g-3p | | 3.10E-17 | | hsa-miR-486-2-5p | | 0.001829794 |
| hsa-miR-7977 | | 1.17E-16 | | hsa-miR-18a-5p | | 0.002230264 |
| hsa-miR-7704 | | 1.76E-16 | | hsa-miR-144-5p | | 0.002587421 |
| hsa-miR-27a-5p | | 4.90E-11 | | hsa-miR-6824-3p | | 0.003022069 |
| hsa-miR-19b-1-3p | | 5.10E-10 | | hsa-let-7d-3p | | 0.00315094 |
| hsa-miR-19b-2-3p | | 5.10E-10 | | hsa-miR-1538 | | 0.003179986 |
| hsa-miR-19a-3p | | 1.83E-09 | | hsa-miR-708-5p | | 0.003334658 |
| hsa-miR-3614-5p | | 4.89E-09 | | hsa-miR-4461 | | 0.005696506 |
| hsa-miR-197-3p | | 9.68E-09 | | hsa-miR-5579-3p | | 0.006170198 |
| hsa-miR-29b-2-3p | | 1.32E-07 | | hsa-miR-101-1-3p | | 0.006704948 |
| hsa-miR-29b-1-3p | | 1.60E-07 | | hsa-miR-33a-5p | | 0.006704948 |
| hsa-miR-33b-5p | | 2.20E-07 | | hsa-miR-4492 | | 0.00713634 |
| hsa-miR-424-5p | | 4.96E-07 | | hsa-miR-492 | | 0.00728764 |
| hsa-miR-3178 | | 5.16E-07 | | hsa-miR-3620-5p | | 0.007371758 |
| hsa-miR-29c-3p | | 8.38E-07 | | hsa-miR-29a-3p | | 0.007945077 |
| hsa-miR-4454 | | 1.33E-06 | | hsa-miR-101-2-3p | | 0.008869229 |
| hsa-miR-5096 | | 2.46E-06 | | hsa-miR-200a-3p | | 0.008869229 |
| hsa-miR-3196 | | 4.14E-06 | | hsa-miR-130a-3p | | 0.011756771 |
| hsa-miR-138-1-3p | | 7.96E-06 | | hsa-miR-1304-3p | | 0.013212544 |
| hsa-miR-324-5p | | 7.96E-06 | | hsa-miR-28-3p | | 0.014248228 |
| hsa-miR-92a-1-3p | | 1.56E-05 | | hsa-miR-33b-3p | | 0.015401502 |
| hsa-miR-3615 | | 1.77E-05 | | hsa-miR-222-3p | | 0.015443876 |
| hsa-miR-92a-2-3p | | 3.34E-05 | | hsa-miR-92b-3p | | 0.015548671 |
| hsa-miR-4508 | | 3.66E-05 | | hsa-miR-221-3p | | 0.016807657 |
| hsa-miR-27b-5p | | 3.68E-05 | | hsa-miR-4485-3p | | 0.017737208 |
| hsa-miR-144-3p | | 5.97E-05 | | hsa-miR-378a-5p | | 0.018839819 |
| hsa-miR-671-3p | | 9.97E-05 | | hsa-miR-7706 | | 0.018839819 |
| hsa-miR-455-3p | | 0.0001465 | | hsa-miR-133a-1-3p | | 0.018953244 |
| hsa-miR-27a-3p | | 0.000156901 | | hsa-miR-133a-2-3p | | 0.018953244 |
| hsa-miR-454-5p | | 0.000156901 | | hsa-miR-452-5p | | 0.020000854 |
| hsa-miR-15b-3p | | 0.000184124 | | hsa-miR-195-5p | | 0.021111735 |
| hsa-miR-34a-5p | | 0.000244107 | | hsa-miR-191-3p | | 0.023516832 |
| hsa-miR-130b-5p | | 0.000248192 | | hsa-miR-3182 | | 0.023699276 |
| hsa-miR-142-5p | | 0.000250921 | | hsa-miR-23b-3p | | 0.025893731 |
| hsa-miR-24-2-3p | | 0.000287921 | | hsa-miR-574-3p | | 0.027126623 |
| hsa-miR-24-1-3p | | 0.000291175 | | hsa-miR-125b-1-3p | | 0.028516248 |
| hsa-miR-1469 | | 0.000379352 | | hsa-miR-619-5p | | 0.029242693 |
| hsa-miR-31-3p | | 0.000405709 | | hsa-miR-4449 | | 0.031270407 |
| hsa-miR-130b-3p | | 0.000434729 | | hsa-miR-1285-1-5p | | 0.033566956 |
| hsa-miR-135b-5p | | 0.000550381 | | hsa-let-7a-1-5p | | 0.034815432 |
| hsa-miR-125b-2-5p | | 0.000622108 | | hsa-let-7a-3-5p | | 0.034815432 |
| hsa-miR-4505 | | 0.000694381 | | hsa-let-7a-2-5p | | 0.035240406 |
| hsa-miR-92a-1-5p | | 0.000751224 | | hsa-miR-1468-5p | | 0.03593155 |
| hsa-miR-125b-1-5p | | 0.000758115 | | hsa-miR-6807-5p | | 0.036508431 |
| hsa-miR-193b-3p | | 0.000775171 | | hsa-miR-1244-1 | | 0.039178038 |
| hsa-miR-1260a | | 0.000794671 | | hsa-miR-1244-2 | | 0.039178038 |
| hsa-miR-141-3p | | 0.000794671 | | hsa-miR-1244-3 | | 0.039178038 |
| hsa-miR-1260b | | 0.000874552 | | hsa-miR-1244-4 | | 0.039178038 |
| hsa-miR-152-3p | | 0.000917998 | | hsa-miR-582-5p | | 0.039984275 |
| hsa-miR-5585-3p | | 0.00098859 | | hsa-miR-107 | | 0.041190974 |
| hsa-miR-362-3p | | 0.001325633 | | hsa-miR-18b-5p | | 0.041363474 |
| hsa-miR-486-1-3p | | 0.001373442 | | hsa-let-7g-3p | | 0.043036788 |
| hsa-miR-451a | | 0.001382208 | | hsa-miR-374b-5p | | 0.043036788 |
| hsa-miR-486-1-5p | | 0.001447144 | | hsa-miR-22-5p | | 0.046420825 |
| ***PKC sample*** | | | | | | |
| hsa-miR-146a-5p | 3.83E-10 | | hsa-miR-30e-5p | | 0.021587918 | |
| hsa-miR-7704 | 4.18E-08 | | hsa-miR-34a-5p | | 0.022264237 | |
| hsa-miR-4485-3p | 2.29E-05 | | hsa-miR-92a-2-3p | | 0.022264237 | |
| hsa-miR-7641-1 | 2.29E-05 | | hsa-miR-145-3p | | 0.023813809 | |
| hsa-miR-7641-2 | 2.29E-05 | | hsa-miR-210-3p | | 0.023813809 | |
| hsa-miR-200b-3p | 2.57E-05 | | hsa-miR-30d-5p | | 0.023813809 | |
| hsa-miR-107 | 3.84E-05 | | hsa-miR-3614-5p | | 0.023813809 | |
| hsa-miR-4492 | 0.000136133 | | hsa-miR-4284 | | 0.023813809 | |
| hsa-miR-30c-1-5p | 0.000290691 | | hsa-miR-4516 | | 0.023813809 | |
| hsa-miR-30c-2-5p | 0.000290691 | | hsa-miR-451a | | 0.023813809 | |
| hsa-miR-379-5p | 0.001166899 | | hsa-miR-1248 | | 0.027020655 | |
| hsa-miR-1273g-3p | 0.002867151 | | hsa-let-7d-3p | | 0.028016858 | |
| hsa-miR-4792 | 0.00382329 | | hsa-miR-24-1-3p | | 0.028016858 | |
| hsa-miR-4508 | 0.005899175 | | hsa-miR-24-2-3p | | 0.028016858 | |
| hsa-miR-193b-3p | 0.006081344 | | hsa-miR-4454 | | 0.028016858 | |
| hsa-miR-145-5p | 0.007233267 | | hsa-miR-10b-5p | | 0.03398129 | |
| hsa-miR-19b-1-3p | 0.011833204 | | hsa-miR-665 | | 0.03398129 | |
| hsa-miR-5096 | 0.011833204 | | hsa-miR-130b-5p | | 0.039063257 | |
| hsa-miR-19b-2-3p | 0.013336474 | | hsa-miR-138-2-5p | | 0.039063257 | |
| hsa-miR-92a-1-3p | 0.013772451 | | hsa-miR-138-1-5p | | 0.046637143 | |

**
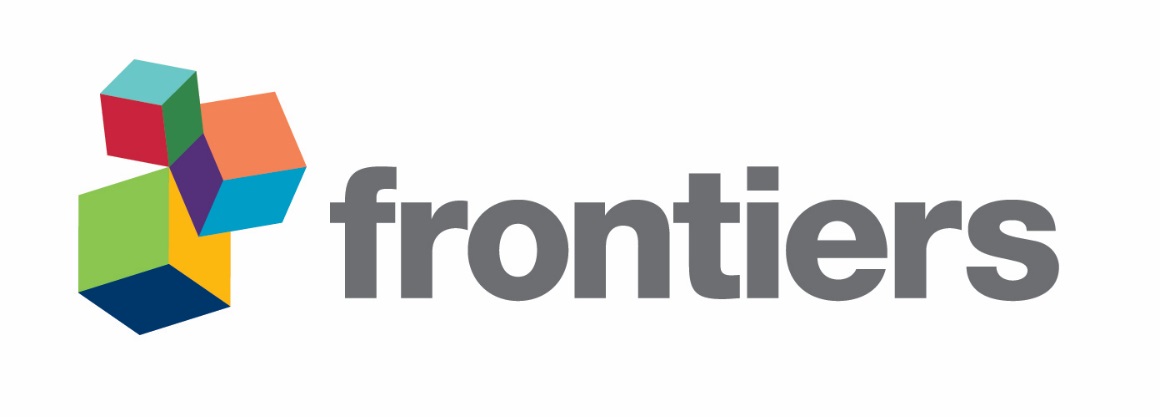
**
